# Anti-*Cryptosporidium* efficacy of BKI-1708, an inhibitor of *Cryptosporidium* calcium-dependent protein kinase 1

**DOI:** 10.1371/journal.pntd.0013263

**Published:** 2025-07-30

**Authors:** Ryan Choi, Matthew A. Hulverson, Deborah A. Schaefer, Dana P. Betzer, Michael W. Riggs, Wenlin Huang, Vicky Sun, Grant R. Whitman, Molly C. McCloskey, Kennan Marsh, Wayne R. Buck, David S. Wagner, Junhai Yang, Andrew P. Bowman, Rita Ciurlionis, Jubilee Ajiboye, Andrew Hemphill, Dilep K. Sigalapalli, Samuel L.M. Arnold, Lynn K. Barrett, Kayode K. Ojo, Erkang Fan, Wesley C. Van Voorhis

**Affiliations:** 1 Department of Medicine, Division of Allergy and Infectious Diseases, Center for Emerging and Re-emerging Infectious Disease (CERID), University of Washington, Seattle, Washington, United States of America; 2 School of Animal and Comparative Biomedical Sciences, College of Agriculture and Life Sciences, University of Arizona, Tucson, Arizona, United States of America; 3 Department of Biochemistry, University of Washington, Seattle, Washington United States of America; 4 Department of Pharmaceutics, University of Washington, Seattle, Washington United States of America; 5 Research and Development, AbbVie Inc, North Chicago, Illinois, United States of America; 6 Cellular, Molecular and Biomedical Sciences Graduate Program, University of Vermont, Burlington, Vermont United States of America; 7 Institute of Parasitology, Vetsuisse Faculty, University of Bern, Bern, Switzerland; University of North Carolina at Chapel Hill, UNITED STATES OF AMERICA

## Abstract

**Background:**

Diarrheal pathogens, such as *Cryptosporidium,* impose a heavy burden of disease in resource-limited regions. Cryptosporidiosis often causes chronic infection in immunocompromised people and gastrointestinal injury in malnourished children, leading to wasting, stunting, and cognitive impairment. Current treatment for cryptosporidiosis fails in these vulnerable populations, highlighting the need for new medicines. Here we describe the anti-*Cryptosporidium* efficacy, pharmacokinetics, and safety of a bumped kinase inhibitor BKI-1708. BKI-1708 inhibits the essential molecular target, calcium-dependent protein kinase 1 (CDPK1), which is highly expressed in the major proliferative stages of the parasite life cycle.

**Methods and Findings:**

Efficacy was demonstrated in the *Cryptosporidium parvum* IFNγ-KO mouse infection and calf diarrhea models. Dose response in the mouse model demonstrated oral doses as low as 15 mg/kg administered daily for 3 days completely suppressed oocyst shedding. Metabolite profiling in pre-clinical species and human hepatocytes identified an active metabolite, M2, which retains sub-micromolar activity against *C. parvum*. Pharmacokinetic analysis of BKI-1708 and M2 in mice demonstrates good systemic exposure, important for treating biliary and upper respiratory infections in some cases of cryptosporidiosis. In mice, M2 reaches 7-fold and >3-fold higher levels over BKI-1708 in plasma and the gastrointestinal tract, respectively. Oral administration of M2 completely suppressed oocyst shedding in the mouse model at doses as low as 8 mg/kg for 3 days. Wide safety margins are demonstrated in mice, rats, and dogs.

**Conclusions:**

BKI-1708 has characteristics of a safe and effective drug for treating *Cryptosporidium* infections in animal models and shows promise for use in humans. Moreover, BKI-1708 and M2 formed in vivo, offer an attractive prospect of a dually active preclinical candidate for the treatment of cryptosporidiosis.

## Author summary

Cryptosporidiosis is a diarrheal disease caused by the parasite *Cryptosporidium*. Symptoms can be long lasting and severe, particularly in malnourished children and immunocompromised people, leading to prolonged gut inflammation, wasting, and chronic infections. Unfortunately, the sole treatment on the market appears to be effective only in people with healthy immune systems. Hence, there is an urgent need for a new and safe drug that works in all patients, one that offers rapid relief from diarrhea and leads to a cure. In this study, we show that a promising drug candidate, BKI-1708, when administered at a low dose in mouse and newborn calf models of *Cryptosporidium* infection, leads to significant reduction in parasite levels in stool. In calves, BKI-1708 quickly resolves diarrhea and leads to improved clinical outcomes. We show BKI-1708 is processed in the liver to form a metabolite that is still potent against *Cryptosporidium*. In mice this metabolite remains in the body for a longer time, extending BKI-1708’s therapeutic potential. Finally, we demonstrate BKI-1708 is well-tolerated at high concentrations in rats and dogs, which considering its substantial efficacy at low concentrations, offers a very promising safety margin during treatment. Thus, our results support further clinical development of BKI-1708 for treatment of human cryptosporidiosis.

## Introduction

Epidemiological studies in the last decade revealed that pathogen-associated diarrheal diseases are a leading cause of death in children under 5 years of age [1,2]. Cryptosporidiosis emerged as a major contributor to global morbidity and mortality in malnourished infants and children under 2 years of age, with an estimated 7.6 million cases of *Cryptosporidium* infections occurring each year in resource-limited populations of Asia and sub-Saharan Africa, and over 200,000 deaths due to moderate-to-severe diarrhea [3,4]. *Cryptosporidium* infections in this population have been associated with prolonged malnutrition, growth stunting, and cognitive impairments, resulting in long-lasting effects on overall development and a loss of 8.2 million disability-adjusted life years (DALYs) [5–9]. *Cryptosporidium* infections can also cause chronic wasting and severe diarrhea in immunocompromised individuals such as those infected with HIV or recipients of organ transplants [10–12]. Furthermore, cryptosporidiosis is a significant public health concern in developed countries, as transmission occurs through the ingestion of infectious *Cryptosporidium* oocysts that are highly resistant to disinfection methods and persist for a long time in the environment, leading to frequent food and waterborne outbreaks and infections through direct contact with animals [13–15].

Current therapeutic options are lacking. Only one drug, nitazoxanide, is approved by the United States Food and Drug Administration for the treatment of cryptosporidiosis. While effective in reducing diarrhea duration by approximately 2 days in immunocompetent individuals [16], nitazoxanide is not approved for use in children <1 year of age, has limited efficacy in malnourished children, and fails to treat AIDS patients [17,18]. Nitazoxanide’s mechanism of action is not well defined, but likely relies on immune system activation for its effect, which may explain its lackluster performance in malnourished and immunocompromised patients [19]. The dearth of treatment options underscores the need for better therapeutics. A new cryptosporidiosis drug should address current unmet needs and demonstrate adequate safety for use in target populations. A target product profile (TPP) was proposed with respect to indication, population, efficacy, and safety to benchmark the ideal and minimum acceptable objectives for a cryptosporidiosis drug development program [20]. An ideal drug should be safe, effective in treating diarrhea and asymptomatic infection in patients of all ages and immune health and pregnancy status, be stable, and be inexpensive to manufacture and distribute.

**Fig 1:**
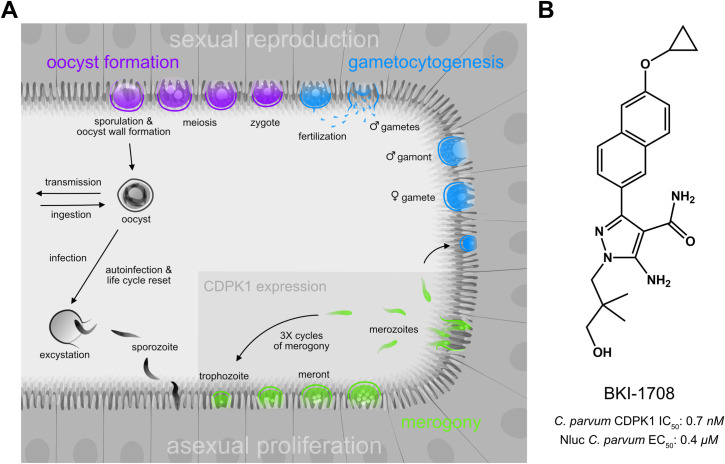
Targeting the *Cryptosporidium* life cycle with BKIs. **(A)** Life cycle of the *Cryptosporidium* parasite. Upon infection, the parasite undergoes 3 rounds of merogeny (green) followed by gametocytogenesis (blue) and oocyst formation (purple). CDPK1, essential for parasite survival, is expressed during rapid asexual proliferation (green). **(B)** BKI-1708 structure and previously reported *C. parvum* CDPK1 IC_50_ and nanoluciferase-expressing (NLuc) *C. parvum* EC_50_ values [29].

Bumped Kinase Inhibitors (BKIs) have shown great therapeutic promise for treating apicomplexan diseases [21–34]. BKIs are ATP-competitive kinase inhibitors. They are named for a particular structural moiety, the “bump”, designed to extend into a hydrophobic pocket within the ATP binding site, a space typically occupied by the gatekeeper residue [26,35–37]. Most mammalian kinases possess a bulky gatekeeper, which limits the size of this pocket and precludes the binding of BKIs. Fortuitously, we discovered that calcium-dependent protein kinase 1 (CDPK1) in *Toxoplasma gondii* and *Cryptosporidium parvum* contained a naturally occurring glycine gatekeeper residue [38,39], which opened up prospects for optimizing BKIs to specifically target these enzymes. CDPKs are serine/threonine kinases unique to plants and apicomplexan parasites and have no closely related orthologs in vertebrates, making them highly attractive drug targets. CDPKs play an important role in motility, invasion, and egress in apicomplexans [40–44]. Recent genetic studies in *C. parvum* revealed that the *cdpk1* gene was refractory to deletion, signifying the essentiality of CDPK1 for *Cryptosporidium* survival [45]. This study also showed that *C. parvum* CDPK1 (*Cp*CDPK1) expression was localized to the asexual stages of the parasite life cycle. Unlike other apicomplexan parasites that require transmission between different host species to complete their life cycles of asexual and sexual reproduction, *Cryptosporidium* completes all developmental stages within the gastrointestinal (GI) tract of a single host in less than 3 days (**Fig 1A**). Upon ingestion of infectious oocysts, sporozoites excyst, penetrate the epithelial layer and develop within intracellular, but extracytosolic parasitophorous vacuoles [46]. The parasite then enters its merogenic stage, a period of rapid proliferation characterized by repeated waves of invasion, asexual replication, and egress from epithelial cells, during which CDPK1 is highly expressed and is indispensable [45] (**Fig 1A**). An elegant series of live imaging experiments recently showed that *C. parvum* adheres to a strict developmental program consisting of 3 successive cycles of merogeny, each lasting 12 h and generating merozoites that egress to start the next wave of invasion [47]. This suggests that each round of asexual amplification offers an early chokepoint for BKIs targeting *Cp*CDPK1.

Several BKIs have strong anti-*Cryptosporidium* activities [25,27–29]. Many candidates fell short of the TPP expectations due to sub-optimal pharmacokinetic/pharmacodynamic (PKPD) profiles and unforeseen toxicity issues [48–50]. However, one preclinical lead, BKI-1708, emerged as a favorable contender for further investigation (**Fig 1B**). Preliminary experiments demonstrated excellent in vitro activity against *Cp*CDPK1 and *C. parvum* parasites and potent in vivo efficacy in an IFNγ-KO mouse model of *Cryptosporidium* infection [29]. Here, we report a comprehensive set of assays characterizing biological activity, metabolism, PKPD, and preclinical safety of BKI-1708 to support its progression to dose finding studies in calves and human trials.

## Methods

### Ethics statement

All animal experiments performed at the University of Washington, USA (Mouse pharmacokinetics (PK) and in vivo efficacy), University of Arizona, USA (in vivo calf efficacy), and University of Bern, Switzerland (mouse pregnancy interference assay), were approved by their respective institutional animal care and use committees (University of Washington IACUC, University of Arizona IACUC, and Animal Welfare Committee of the Canton of Bern, respectively). Animal experiments by AbbVie Inc. (Rat, dog, and monkey PK; Mouse MALDI mass spectrometry imaging; in vivo rat and dog cardiotoxicity; and 5-day rat repeat dose toxicology) and SRI International (14-day rat and 5-day dog repeat dose toxicology) were reviewed and approved by AbbVie IACUC and SRI International IACUC, respectively, and conducted in Association for Assessment and Accreditation of Laboratory Animal Care (AAALAC) accredited facilities. Animals used in these experiments were handled in strict accordance with practices made to minimize suffering and in accordance with the PHS *Guide for the Care and Use of Laboratory Animals*. Calf studies at the University of Arizona were also conducted in compliance with guidelines in the Animal Welfare Act and *Guide for the Care and Use of Agricultural Animals in Research and Teaching* (Federation of Animal Science Societies), in facilities fully accredited by the American Association for Laboratory Animal Care.

### Study design

Study objectives were to characterize the therapeutic potential and safety of BKI-1708 for cryptosporidiosis treatment. In vitro efficacy was evaluated against the target enzyme and several *C. parvum* isolates as well as a second species, *C. hominis*, relevant in human infections. In vivo efficacy was assessed using immunocompromised acute mouse and newborn calf models of *Cryptosporidium* infection. In vitro and in vivo ADME studies identified a potential active metabolite that may be contributing to the potency of BKI-1708. Finally, in vitro and in vivo toxicological studies were conducted to assess preclinical safety and to enable advancement to human clinical trials.

### Previously published methods

Methods for BKI-1708 chemical synthesis [29], in vitro IC_50_ determination against *Cp*CDPK1 and *Hs*SrC [29, 51], EC_50_ determination against *C. parvum* parasites in the Nanoluciferase [28,29,52], and high-content imaging [53] assay systems, efficacy in IFNγ-KO immunocompromised acute mouse model of NLuc *C. parvum* infection [27–29,48], solubility [48,54], permeability [48], plasma protein binding [29], kinome profiling [55], cytotoxicity in CRL-8155 and HepG2 cells [56], zebrafish developmental safety assays [57], the modified µAmes test [58], the in vitro micronucleus test [59], hERG inhibition [60], metabolic stability assays [61,62], CYP phenotyping assays [63], CYP inhibition assays [64], metabolite identification [63,65], mouse PK [29,48,56,66], mouse GI exposure PK [27,48], efficacy in a newborn calf model of *C. parvum* infection [25,67], mouse pregnancy interference assay [57], and in vivo rat and dog cardiotoxicity [68] were previously reported. For EC_50_ determination, HCT-8 host cell lines were infected with *C. parvum* isolates sourced from U AZ Laboratory (Tucson, AZ), Bunch Grass Farms (Deary, ID), or Zambriski Laboratory (Washington State University, WA). For NLuc assays, luminescence was detected using the Nano-Glo Luciferase Assay System (Promega, Madison, WI) and an EnVision Multimode plate reader (PerkinElmer, Inc, Waltham, MA). All other methods are specified in S1 File.

### Statistics

Statistical analyses were performed using Microsoft Excel (Microsoft, Redmond, WA, USA) or GraphPad Prism 6 (GraphPad Inc., La Jolla, CA, USA). In general, sample sizes were small. Animal PK data were collected for n ≥ 3 per group, and SD values are provided for tables with datasets of n > 2. Calf experiment data were collected from multiple cohort trials (n = 16 vehicle control; n = 3, BKI-1708 treated) and error bars on graphs represent the SEM. Control calves were contemporaneous with calves treated with BKI-1708. Mann-Whitney U test of mean AUCs were used to determine significance between test groups.

## Results

### In vitro potential of BKI-1708 against *Cp*CDPK1 and *C. parvum* and *C. hominis* parasites

The inhibitory potency of BKI-1708 was previously reported [29] with sub-nanomolar half maximal inhibitory concentrations (IC_50_) against *Cp*CDPK1 and sub-micromolar half maximal effective concentrations (EC_50_) against excysted nanoluciferase-expressing (NLuc) *C. parvum* parasites (strain background, U AZ Laboratory Isolate, Tucson, AZ) co-cultured with HCT-8 cells (**Fig 1B**). BKI-1708’s in vitro cellular potency was further validated against the California Institute for Biomedical Research (Calibr) (Scripps Research, San Diego, CA) *Cryptosporidium* panel, which utilizes high-content imaging of *Cryptosporidium* proliferation in HCT-8 cells to measure inhibitory activities of test compounds in three calf-derived *C. parvum* isolates: U AZ Laboratory isolate [69,70], Bunch Grass Farms isolate (Deary, ID) [71], and Zambriski Laboratory isolate (Washington State University, WA) [72]; and a gnotobiotic piglet-derived *C. hominis* TU502 isolate (Tufts University Cummings School of Veterinary Medicine, MA) [73,74]. The *C. parvum* isolates were more sensitive to BKI-1708, exhibiting 2–8-fold lower EC_50_s compared to the NLuc strain (**Table 1**). The *C. hominis* TU502 isolate also exhibited sub-micromolar susceptibility, confirming that BKI-1708 is active against both species most relevant to human disease. Since the genomes of these species share high homology (95–97%) with the CDPK1 loci exhibiting 99% sequence identities, this correlation in potency was expected.

**Table 1:** In vitro activity of BKI-1708 against the Calibr “cell-based” *Cryptosporidium* panel.

*Cryptosporidium* strain	EC_50_ (μM)	EC_90_ (μM)
***C. parvum* U AZ Laboratory isolate**	0.09	1.07
***C. parvum* Bunch Grass Farms isolate**	0.21	1.67
***C. parvum* Zambriski Laboratory isolate**	0.05	4.30
***C. hominis* TU502 isolate**	0.65	1.53

### Electron micrographs of *C. parvum* infected HCT-8 cells treated with BKI-1708

**Fig 2:**
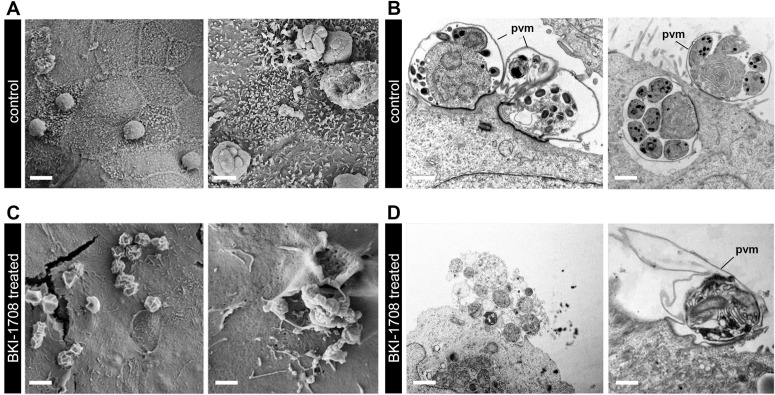
Visualizing the effects of BKI-1708 treatment of *C. parvum* infected monolayers. **(A)** SEM of untreated HCT-8 monolayers infected with *C. parvum*. Right panel shows egressed merozoites. Bar: left, 2.5 μm; right, 1.4 μm. **(B)** TEM of vehicle-treated monolayers show various stages of merogony. pvm: parasitophorous vacuolar membrane. Bar: left, 0.8 μm; right, 0.8 μm. **(C)** SEM of infected monolayers after 24 h treatment with BKI-1708. Bar: left, 2.5 μm; right, 1.6 μm. **(D)** TEM of BKI-1708 treated monolayers. Bar: left, 1.0 μm; right, 1.0 μm.

We used scanning electron microscopy (SEM) and transmission electron microscopy (TEM) to visualize *C. parvum* infected HCT-8 monolayers after 45 h treatment with 2.5 μM BKI-1708 (2.2X EC_90_). Images were captured 48 h post infection (p.i.) and compared to vehicle controls. SEM of controls showed a regular distribution of corpulent vacuoles along the surface of the monolayer, demonstrating a robust infection (**Fig 2A**). In addition, occasional merozoites were observed emerging from their parasitophorous vacuoles to initiate another cycle of invasion. TEM analysis revealed meronts and budding merozoites from early and advanced stages of merogeny enveloped within intact parasitophorous vacuoles, and firmly attached to the surface of the host cell (**Fig 2B**). In stark contrast, SEM of infected monolayers treated with BKI-1708 revealed a sparse population of parasites, and the few identified had parasitophorous vacuoles appearing crenated and barren (**Fig 2C**). Ruptured vacuoles exposed parasites that were underdeveloped and malformed. TEM analysis also revealed a dearth of intact asexual and sexual stage parasites. Only loosely attached, irregular parasite remnants encapsulated in vacuoles lacking well-defined membranes could be identified (**Fig 2D**).

### In vivo efficacy of BKI-1708 in a *C. parvum* IFNγ-KO mouse model

**Fig 3:**
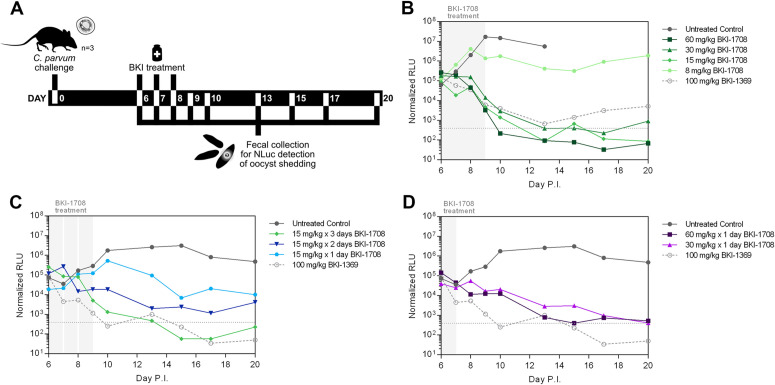
BKI-1708 efficacy in a mouse model of *Cryptosporidium* infection. **(A)** Experimental design of the NLuc *C. parvum* IFNγ-KO mouse model. Mice (n = 3) are orally challenged with 10^4^ NLuc-expressing *C. parvum* oocysts on day 0 and treatment commenced on day 6. Fecal samples are collected regularly until day 20 p.i. for NLuc detection of oocyst shedding. **(B)** Results of 3-day BKI-1708 treatment. The dotted line at 300 RLU denotes the limit of detection (LoD) for this assay. The untreated control group (dark gray closed circle, solid line) in this experiment were euthanized on day 13 p.i. due to excessive weight loss and moribundity. The control (light gray open circle, dashed line) used for all mouse efficacy experiments was 100 mg/kg BKI-1369 administered for 5 days, a former late-lead candidate for cryptosporidiosis [27]. **(C)** Results of BKI-1708 administered at 15 mg/kg for 1, 2, and 3 days. **(D)** Results of single dose BKI-1708 treatments.

In vivo efficacy of BKI-1708 was previously demonstrated [29] in an IFNγ-KO immunocompromised acute mouse model of *Cryptosporidium* infection with 5 days of once daily (QD) oral doses as low as 8 mg/kg. Plasma levels sampled after the 4^th^ dose reached peak steady-state concentrations of 2.6 ± 0.7 μM and oocyst shedding remained suppressed with >3 log reduction over untreated controls at 20 days p.i., the minimum threshold for effective clearance in this model. Encouraged by these results, we investigated a truncated dosing period of 3 days (**Fig 3A**). Mice were orally infected with 10^4^ NLuc-expressing *C. parvum* oocysts (strain background, U AZ Laboratory Isolate, Tucson, AZ) and treated with 60, 30, 15, and 8 mg/kg QD doses beginning day 6 p.i. (**Fig 3B**). Fecal analysis revealed that by day 10 p.i. the 60 mg/kg treated mice had completely suppressed oocyst shedding to levels below the limit of detection (LoD) and peak plasma concentrations reached 13.2 ± 2.5 μM. The oocyst clearance rate for the 3-day regimen was delayed compared to the 5-day but resulted in an equivalent level of suppression by day 9 p.i. [29]. The 30 mg/kg and 15 mg/kg groups were similarly delayed, reaching the LoD for oocyst shedding by day 13 p.i. and peak plasma concentrations of 6.9 ± 4.2 μM and 5.3 ± 1.7 μM, respectively (S1 Table). Nevertheless, these dose groups performed equally well in suppressing oocyst shedding up to 20 days p.i., with >4 log reductions compared to peak infection levels of the untreated control group, which had to be euthanized on day 13 p.i. due to excessive weight loss and moribundity. Whereas 8 mg/kg dosed for 5 days was successful in suppressing oocyst shedding in previous experiments, a 3-day regimen at this dose level failed to recapitulate efficacy. Steady-state plasma concentrations of the 8 mg/kg group reached 3.3 ± 1.5 μM (S1 Table), congruent with peak levels obtained with the efficacious 5-day regimen.

Next, we considered whether further reductions in dosing frequency would be achievable. As 15 mg/kg x 3 days worked well to control infection, we evaluated whether 2-day or single dose regimens would be sufficient. A 15 mg/kg x 2 day treatment resulted in a 3.1 log reduction in oocyst shedding by day 13 p.i. and a 2.1 log reduction by day 20 p.i. compared to untreated animals (**Fig 3C**). Although significant overall reduction in oocyst shedding was observed, this treatment did not meet the threshold of >3 log reduction by day 20 p.i. to be considered a successful regimen. Furthermore, a single 15 mg/kg dose resulted in 1.4 log and 1.7 log reductions at 13 and 20 days p.i., respectively, which also fell short of the threshold for efficacy. However, a single dose of 60 mg/kg resulted in log reductions of 3.5 and 3.0 at 13 and 20 days p.i., respectively (**Fig 3D**). Similarly, a single 30 mg/kg dose produced 3.0 log reductions at 13 and 20 days p.i. It is notable that these single dose regimens resulted in a gradual reduction of oocysts compared to the relatively rapid rate of clearance observed with repeated dose regimens. In summary, it appeared that single dose and 2-day treatment regimens of BKI-1708 were suboptimal for complete and rapid resolution, and 3 days of 15 mg/kg daily oral dosing was optimal for efficacy.

### In vitro safety profile of BKI-1708

Salt and polymorph screens were conducted to identify the most stable solid-state form of BKI-1708 for further development stages. The same crystalline free base anhydrate that was synthesized at onset of the project was identified to be the most stable physical form. This polymorph was found to be non-hygroscopic and highly stable, with bulk stability under storage conditions of up to 60°C and relative humidity of up to 95% for 2 weeks.

Several assays were employed to assess the selectivity and safety of BKI-1708 in vitro. BKI-1708 had high passive permeability across MDCK cell monolayers (P_appA__→__B_: 33.8x10^-6^ cm/s; P_appB__→__A_: 28x10^-6^ cm/s), low solubility in PBS and fasted-state simulated intestinal fluids (18 and 38 μM, respectively), and moderately higher solubility in simulated gastric fluids and fed-state simulated intestinal fluids (58 and 89 μM, respectively). The blood to plasma ratio was 0.84, suggesting that BKI-1708 is not preferentially sequestered in red blood cells. Plasma protein binding (PPB) was measured via equilibrium dialysis and was determined to be substantially bound in various species: mice (96%), rat (95%), dog (97%), monkey (94%), human (96%), and calf (97%). This may be beneficial from a safety standpoint, as unbound BKI-1708 in plasma will be in equilibrium with free BKI-1708 in tissues and the high degree of PPB may reduce the potential for BKI-1708 to engage liability targets.

We screened a representative panel of 80 + human kinases to characterize the kinome liabilities of BKI-1708. BKI-1708 exhibited <1 μM IC_50_s for MEK1, RIPK2, and PKD3 (**Table 2**). To further elucidate the inhibitory potential for MEK1, which regulates tumor cell proliferation, we tested BKI-1708 in a cellular proliferation assay in 4 carcinoma cell lines (A-375, Hs294T, MEWO, and Colo678) against a clinically approved MEK inhibitor, Trametinib [75,76]. BKI-1708 was inactive up to 1 μM in these assays and exhibited an IC_50_ >3.6 μM, a >294-fold difference in activity compared to Trametinib (S1 Fig). Thus, MEK1 inhibition is unlikely to be a safety issue with BKI-1708. Furthermore, BKI-1708 was tested for anti-proliferative activities against CRL-8155 B-lymphocyte and HEPG2 hepatocyte cell lines and resulted in half maximal cytotoxic concentrations (CC_50_s) of >80 μM for both cell lines.

**Table 2:** In vitro kinase liability profile of BKI-1708.

Kinase	IC_50_ (μM)*
**MEK1**	0.06
**PKD3**	0.14
**RIPK2**	0.88
**MST1**	1.09
**STK33**	2.3
**EGFR**	2.53
**DDR1**	2.71
**MAP4K1**	4.07
**ALK**	4.33
**Flt1**	4.59
**Fyn**	5.04
**MAP4K4**	5.34
**Aurora2**	5.4
**RET**	6.92
**CSK**	7.9

*Only kinases with activity <10 μM listed

Next, we tested BKI-1708 against a bioprofiling panel of 20 liability targets including G protein-coupled receptors (GPCRs) and ion channels in both agonist and antagonist readouts, and biochemical functional assays for nuclear hormone receptors and phosphodiesterases. All assays were inactive at 10 μM concentrations (S2 Table). We also submitted BKI-1708 for supplemental screening in the Cerep (Eurofins, France) assay against a panel of 71 common liability targets consisting of receptors, transporters, ion channels, and various oxidases (S3 and S4 Tables). All assays were inactive at 10 μM concentrations, except PPARУ. However additional tests found the IC_50_ to be >100 μM, suggesting that the initial hit was a false positive (S2 Fig).

### Other in vitro safety assessments

As a screen for teratogenicity, freshly fertilized zebrafish (*Danio rerio*) eggs were exposed to BKI-1708. Embryo development was microscopically monitored over 4 days, after which the rate of malformations and embryo deaths were scored against vehicle controls. BKI-1708 did not visibly affect zebrafish embryo development up to 2 μM. BKI-1708 was also subjected to mutagenicity tests including the microAmes (μAmes) test to assess its ability to induce reverse mutations in *Salmonella* bacteria, and the in vitro micronucleus (IVMN) test to detect the presence of micronuclei in interphase cells as a measure of drug-induced chromosomal damage. No dose-dependent revertant colonies were detected in the μAmes test and no change in the frequency of micronucleated cells was observed in the IVMN test, suggesting low potential for mutagenicity and genotoxicity.

The cardiovascular (CV) safety profile of BKI-1708 was previously reported as exhibiting 12–13 µM IC_50_ hERG inhibition in thallium flux and patch clamp (Qpatch) assays [29,49] (**Table 3**). Additionally, an in vitro QT-Inotropy screening assay (QTiSA) was employed to evaluate direct compound effects on cardiac contractility, effective refractory period (ERP) prolongation (repolarization-QT liability), and cardiac sodium excitability in isolated rabbit ventricular myocytes. BKI-1708 was tested at 1, 3, 10, and 30 μM, but did not produce appreciable changes in contractility, ERP, or excitability at the concentrations tested (**Table 3**).

**Table 3:** In vitro cardiovascular safety profile of BKI-1708.

hERG (Tl flux)	hERG (Qpatch)	Change in contractility (%)			Change in ERP (ms)			Change in threshold excitability (V/cm)		
IC_50_ (μM)	IC_50_ (μM)	IC_50_ (μM)	MSC (μM)	Change (%)	IC_50_ (μM)	MSC (μM)	Change (%)	IC_50_ (μM)	MSC (μM)	Change (%)
13	12.3	>30	>30.1	-12.1	>30.1	>30.1	-1.9	>30.1	>30.1	0.1

MSC: minimum significant concentration; ERP: effective refractory period. A change of >15% is considered to be significant.

### In vitro metabolite profiling of BKI-1708

BKI-1708 was incubated in pooled liver microsomes (LM) from various pre-clinical species with NADPH cofactor to determine its metabolic stability. The depletion rate for BKI-1708 appeared to be highest in mouse LM with a 19 min half-life (t_1/2_) (**Table 4**). Next, we conducted reaction phenotyping assays with 7 common recombinant human cytochrome P450 (CYP) enzymes (1A2, 2B6, 2C8, 2C9, 2D6, 2C19, and 3A4) to identify their relative contributions to the metabolism of BKI-1708. CYP3A4 appeared to be the primary metabolizer (83.7% contribution), followed by CYP1A2 and CYP2B6 (**Table 5**). The remaining CYP enzymes appeared to have negligible effects on BKI-1708 stability. We then evaluated the propensity of BKI-1708 to inhibit CYP enzymes, which may potentiate drug-drug interactions for co-administered medications. A decrease in metabolite formation from probe substrates compared to controls was used to estimate IC_50_s for each CYP enzyme. The IC_50_s for the 7 common CYP enzymes were >10 μM except CYP2C9, which was 9.6 μM (**Table 5**). In addition, we examined whether BKI-1708 induces CYP3A4 expression, as the apparent contribution of this CYP isoform to BKI-1708 metabolism was highest. Induction was assessed at 10 μM in primary human hepatocytes and CYP3A4 mRNA expression was measured by qRT-PCR. Incubation with BKI-1708 did not result in a significant increase in mRNA expression compared to vehicle control.

**Table 4:** In vitro stability of BKI-1708.

	Liver microsome (LM) stability			Hepatocyte stability			
Species	CL_int_ (µL/min/mg)	CL_int_scaled_ (L/h/kg)	t_1/2_ (min)	CL_int_ (µL/min/10^6^ cells)	CL_int_scaled_ (L/h/kg)	t_1/2_ (min)	LM:hepatocyte CL_int_scaled_ ratio
**Mouse**	143.0	27.4	19	5.2	2.6	345	10.5
**Rat**	29.0	3.1	96	15.8	4.6	88	0.7
**Dog**	41.1	3.6	68	6.8	1.6	203	2.3
**Monkey**	35.2	2.4	79	9.1	1.6	152	1.5
**Human**	31.5	2.0	88	17.7	3.1	79	0.7
**+ CYP3A4i**	ND	ND	ND	12.3	2.1	113	
**+ pan-CYPi**	ND	ND	ND	1.7	0.3	836	

CL_int_: in vitro intrinsic clearance; CL_int_scaled_: scaled intrinsic clearance; t_1/2_: half-life; CYP3A4i: incubations with Azamulin CYP3A4 inhibitor; pan-CYPi: incubations with 1-ABT pan-CYP inhibitor and tienilic acid (CYP2C9 inhibitor)

**Table 5:** CYP reaction phenotyping and inhibition profiles of BKI-1708.

	CYP phenotyping				CYP inhibition	
CYP isoform	CL_int_ (µL/min/pmol)	CL_int_scaled_ (L/h/kg)	t_1/2_ (min)	% contribution		IC_50_ (µM)
**1A2**	0.033	0.048	208	9.6		147.0
**2B6**	0.079	0.038	88	6.6		52.0
**2C8**	<0.029	<0.019	>240	0.0		22.8
**2C9**	NV	NV	NV	0.0		9.6
**2D6**	<0.029	<0.019	>240	0.0		17.1
**2C19**	<0.029	<0.019	>240	0.0		33.5
**3A4**	0.33	0.706	21	83.7		>250; 22.5

CYP phenotyping performed using recombinant supersomes. CYP inhibition determined in human microsomes. CYP3A4 IC_50_ values using midazolam and testosterone substrates, respectively.

We evaluated the metabolic stability of BKI-1708 in primary hepatocytes from several pre-clinical species as these cells contain the full complement of liver metabolizing enzymes including cytosolic phase I enzymes (esterases, oxidases, hydrolases, etc.) and phase II conjugating enzymes (sulfotransferases, acetyltransferases, glucuronosyltransferases, etc.). Comparing the stability of BKI-1708 in LM and hepatocyte incubations, the LM:hepatocyte scaled intrinsic clearance (CL_int_scaled_) ratio for mouse was high (**Table 4**) and may suggest BKI-1708 uptake into mouse hepatocytes is rate-limiting and the major metabolic pathway may be CYP-mediated [77,78]. The metabolic stability in human hepatocytes was additionally assessed in the presence of Azamulin, a selective CYP3A4 inhibitor, and a pan-CYP inactivator cocktail (1-ABT + tienilic acid). With CYP3A4 inhibited, the t_1/2_ increased from 79 to 113 min and the fraction metabolized (f_m_) by CYP3A4 was estimated to be 0.31. However, with pan-CYP inactivation, the t_1/2_ increased by >10 fold to 836 min and the f_m_ by all CYP enzymes was calculated to be 0.91. These results suggest that while CYP3A4 may contribute significantly to metabolism, it may not be the primary CYP enzyme responsible for hepatic clearance of BKI-1708 in humans. It is likely that the CYP enzyme in question was not part of the panel of 7 common enzymes tested in the phenotyping assay. Further investigation of other CYP isoforms will be necessary to identify the major metabolizer of BKI-1708.

**Fig 4:**
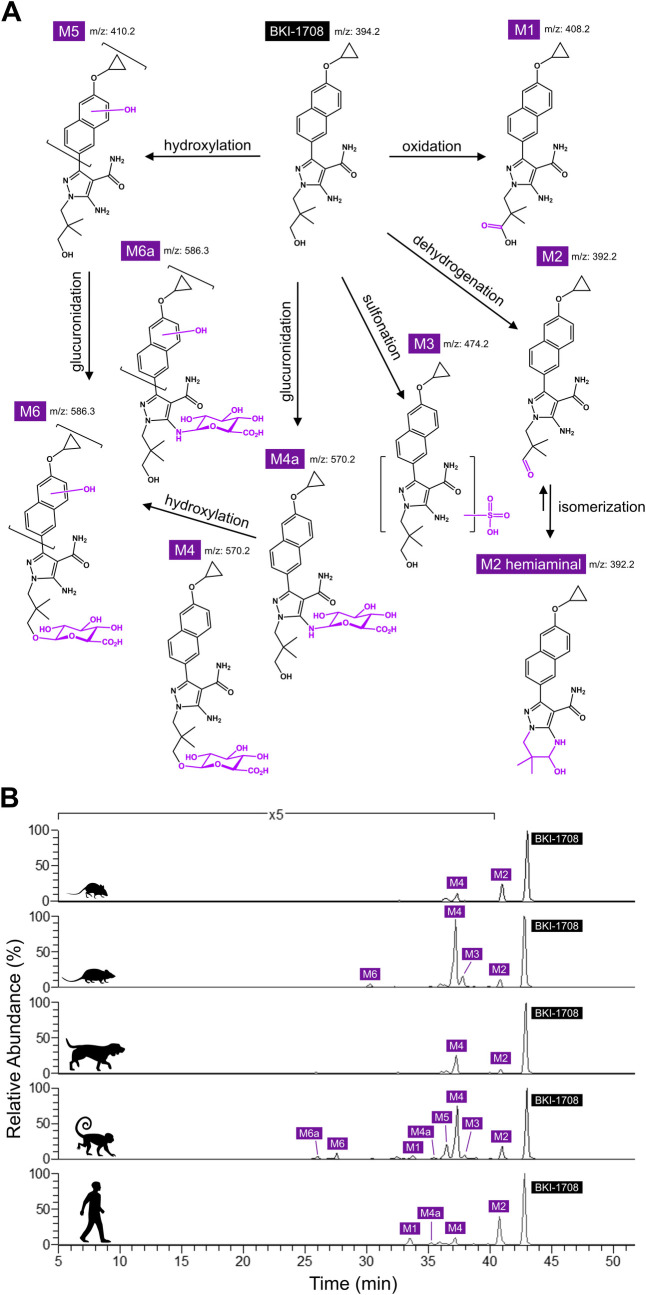
Metabolite identification in primary hepatocytes. **(A)** Probable biotransformation products of BKI-1708 determined via incubation in cross-species hepatocytes. Dehydrogenation and O-glucuronidation appear to be the main metabolism pathways. The dehydrogenation product, M2, undergoes isomerization to form a more stable, cyclized hemiaminal. **(B)** Ion chromatograms showing relative abundance of biotransformation products from cross-species hepatocytes. Top to bottom, results are from mouse, rat, dog, monkey, and human hepatocyte incubations. M2 appeared to have the highest abundance in mouse and human incubations and was synthesized for further characterization.

To determine which metabolites are formed in biological matrices of treated animals, BKI-1708 was incubated with hepatocytes from several preclinical species and humans and biotransformation was analyzed by LC-MS/MS. Eight metabolites were identified in the hepatocyte incubates resulting from metabolic pathways involving oxidation (M1), dehydrogenation (M2), sulfonation (M3), glucuronidation (M4, M4a), and hydroxylation (M5, M6, M6a) (**Fig 4A**). Dehydrogenation of the terminal alcohol of BKI-1708 results in the formation of M2, which possesses a terminal aldehyde group that can reversibly react with the neighboring amino group at position 5 of the pyrazole ring to form a stable cyclized hemiaminal. M2 was detected in all tested species but appeared to be the primary metabolite with the greatest relative abundance in mice and human hepatocytes (**Fig 4B**). The glucuronidation product, M4, was also detected in all species, but showed higher relative abundance in rats, dogs, and monkeys, suggesting a divergence in the metabolic pathways in these species compared to mice and humans. The M3 sulfonation product and M6 glucuronide were only formed in rat and monkey hepatocytes and the M1 oxidation product was only present in primate hepatocytes.

### In vitro profile of BKI-1708 metabolite, M2

As M2 appeared to be the predominant metabolite in mice and human hepatocytes, it was synthesized for assessment of its safety in vitro. The aldehyde and hemiaminal isomers of M2 share the same mass and can interconvert, making efforts to produce purely one isomer quite challenging. Distinguishing isomers using standard C18 reverse-phase LC-MS have proven to be difficult as they appear at the same retention time and give identical mass spectra. Attempts at optimization using normal-phase LC-MS were also unsuccessful. While we were unable to generate the pure aldehyde, we optimized the synthetic route to predominately produce the hemiaminal, as confirmed by thin layer chromatography (TLC). BKI-1708 was used as starting material and the yield was low (15%). The hemiaminal was stable in pH 6.8 phosphate buffer at ambient temperature for up to 24 h, but it was found to interconvert to a mixture of the aldehyde and the hemiaminal upon extended storage.

Like BKI-1708, the PPB of M2 was high in mice (96%), rat (94%), dog (90%), human (96%), and calf (95%) plasma. The off-target kinase profile was clean, with no activity against the small-gatekeeper SrC (IC_50_ >10 μM). Only PKD3 exhibited an IC_50 _<1 μM (0.23 μM IC_50_) in the 80 kinome panel. However, M2 showed slightly higher inhibition compared to BKI-1708 in the hERG patch clamp assay with an IC_50_ of 6.2 μM. M2 was also screened against the Cerep panel (S5 and S6 Tables) and results showed IC_50_s >10 μM for all targets except for AT_2_ agonism (4.6 μM) (S5-S7 Figs). Furthermore, some cytotoxicity was observed in CRL-8155 and HepG2 cells with 15.6 μM and 13.4 μM CC_50_s, respectively. Next, M2 was exposed to zebrafish embryos to screen for teratogenic effects and was found to be safe up to 20 μM. Finally, M2 was negative in both the μAmes and IVMN mutagenicity tests.

M2 was highly stable in rat, dog, and human LMs and least stable in monkey LMs (**Table 6**). Hepatocyte incubations resulted in similar t_1/2_s for dog, monkey, and human, while mouse and rat showed faster rate of reduction. Reaction phenotyping assays revealed CYP3A4 and CYP1A2 to be the main contributors of M2 metabolism (**Table 7**). CYP inhibition studies were conducted for inhibition of CYPs 1A2, 2C9, and 3A4 in human LM. M2 did not show any inhibition up to 40 μM concentrations for these isoforms.

**Table 6:** In vitro stability of BKI-1708 metabolite, M2.

	Liver microsome (LM) stability			Hepatocyte stability			
Species	CL_int_ (µL/min/mg)	CL_int_scaled_ (L/h/kg)	t_1/2_ (min)	CL_int_ (µL/min/10^6^ cells)	CL_int_scaled_ (L/h/kg)	t_1/2_ (min)	LM:hepatocyte CL_int_scaled_ ratio
**Mouse**	23.6	4.5	118	5.3	2.7	262	1.7
**Rat**	<23.1	<2.5	>120	13.2	3.8	105	<0.7
**Dog**	<23.1	<2	>120	3.4	0.8	411	<2.5
**Monkey**	48.6	3.3	57	3.6	0.6	389	5.5
**Human**	<23.1	<1.5	>120	3.7	0.6	376	<2.5

CL_int_: in vitro intrinsic clearance; CL_int_scaled_: scaled intrinsic clearance; t_1/2_: half-life.

**Table 7:** CYP reaction phenotyping and inhibition profiles of BKI-1708 metabolite, M2.

	CYP phenotyping				CYP inhibition
CYP isoform	CL_int_ (µL/min/pmol)	CL_int_scaled_ (L/h/kg)	t_1/2_ (min)	% contribution	IC_50_ (µM)
**1A2**	0.115	0.167	60	47.0	>40
**2B6**	0.044	0.021	159	5.1	ND
**2C8**	<0.029	<0.107	>240	0.0	ND
**2C9**	<0.029	<0.107	>240	0.0	>40
**2D6**	<0.029	<0.011	>240	0.0	ND
**2C19**	<0.029	<0.007	>240	0.0	ND
**3A4**	0.141	0.301	49	47.9	>40

CYP phenotyping performed using recombinant supersomes. CYP inhibition determined in human microsomes; ND: data not available.

Next, we considered whether M2 had retained activity against *Cp*CDPK1 and efficacy against *C. parvum* parasites. An IC_50_ of 92.5 nM was determined against *Cp*CDPK1, representing a 132-fold reduction in potency compared to BKI-1708. However, when tested against NLuc *C. parvum* parasites, activity was sustained at the sub-micromolar level (**Table 8**). Additionally, the Bunch Grass Farms *C. parvum* and TU502 *C. hominis* isolates from the Calibr *Cryptosporidium* panel showed comparable sub-micromolar susceptibilities. These results suggest that M2 could be contributing to BKI-1708’s excellent in vivo potency in mice.

**Table 8:** In vitro activity of BKI-1708 metabolite, M2.

*Cryptosporidium* strain	IC_50_ or EC_50_ (μM)	EC_90_ (μM)
*C. parvum* CDPK1 enzyme*	0.0925	ND
**NLuc *C. parvum* (UW)**	0.73	ND
**NLuc *C. parvum* (Calibr)**	0.34	1.24
***C. parvum* Bunch Grass Farms isolate**	0.32	0.77
***C. hominis* TU502 isolate**	0.44	1.67

*IC_*50*_ values for *C. parvum* CDPK1.

NLuc *C. parvum* EC50 values from assays performed at University of Washington (UW) and Calibr; ND: data not available.

### Systemic exposure and gastrointestinal presence of BKI-1708 and M2 in mice

BKI-1708 was orally administered (PO) to Balb/c mice at 15 mg/kg to quantify plasma exposures of BKI-1708 and formation of M2. Peak plasma concentrations (C_max_) of BKI-1708 reached 10.8 ± 1.8 μM by 40 min with a t_1/2_ of 106 min (**Table 9**). M2 reached a C_max_ of 13.1 ± 1.7 μM by approximately 120 min, surpassing levels by BKI-1708. Area-under-curve (AUC) ratios of metabolite:parent molecule (M/P) reveal 3.4-fold higher systemic exposures of M2. BKI-1708 was then administered intravenously (IV) to Balb/c mice via retro-orbital injection at a dose of 3 mg/kg. The volume of distribution (V_d_) and clearance in plasma (CL_p_) were moderate (**Table 9**), with an oral bioavailability (%F) of 93%. Next, we administered M2 directly to Balb/c mice via PO and IV routes to determine its PK profile. A 30 mg/kg PO dose resulted in a %F of 43%. A 1 mg/kg IV dose revealed that M2 had lower V_d_ and CL_p_ compared to BKI-1708 (**Table 9**).

**Table 9:** Pharmacokinetic profile of BKI-1708 and M2 in mice.

Mouse Strain (Compound Administer-ed)	Route	Dose (mg/kg)	C_max_ (µM ± SD)	C_0_ (µM ± SD)	T_max_ (min)	t_1/2_ (min)	AUC_0-inf_ (min*µmol/L ± SD)	CL_p_ (mL/min/kg)	V_d_ (L/kg)	%F	M2 C_max_ (µM ± SD)	M2 T_max_ (min)	M2 t_1/2_ (min)	M2 AUC_0-inf_ (min*µmol/L ± SD)	M/P AUC
**Balb/c (BKI-1708)**	**PO**	**15**	10.8 ± 1.8		40	106	1835 ± 713			93	13.1 ± 1.7	120	183	6445 ± 654	3.4
	**IV**	**3**		5.4 ± 0.3		57	395 ± 36	17.7	1.5		4.3 ± 0.1	120	117	988 ± 162	2.9
**Balb/c (M2)**	**PO**	**30**	13.1 ± 1.3		90	184	5185 ± 1500			43					
	**IV**	**1**		4.2 ± 1.3		106	400 ± 42	6.2	0.9						
****IFN** **γ** **-KO BL6 (BKI-1708)****	**PO**	**15**	4.5 ± 1.3		50	86	864 ± 592			100	11.6 ± 3.5	120	195	5948 ± 1398	6.9
	**IV**	**3**		4.4 ± 2.9		41	170 ± 25	45.5	2.7		2.6 ± 0.6	120	158	792 ± 18	4 .7

PO: oral; IV: intravenous; C_max_: peak concentration; C_0_: initial concentration; T_max_: time at peak concentration; AUC_0-inf_: area-under-curve extrapolated to infinity; CL_p_: plasma clearance; V_d_: volume of distribution; %F: oral bioavailability; M/P AUC: ratio of metabolite to parent AUCs.

**Fig 5:**
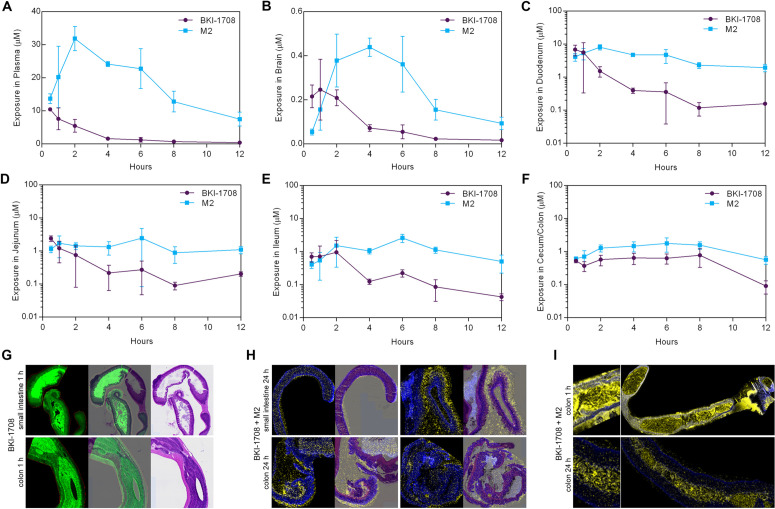
In vivo exposure of BKI-1708 in mice. **(A)** Plasma exposure of BKI-1708 and M2 after 30 mg/kg PO administration of BKI-1708 in Balb/c mice (n = 3 per time point). **(B)** Similar to plasma, exposures from homogenized brain tissue show M2 accumulating to levels >3-fold higher than the parent molecule. **(C-F)** Exposure in GI sections reveal BKI-1708 levels are highest within the first 2 h post administration while M2 levels build up to 6 h and remain at or above 1 μM. (**G**) timsTOF MALDI-MSI showing strong BKI-1708 presence (green) in mouse small intestine and colon sections 1 h post BKI-1708 administration (n = 4). Signals were overlayed on hematoxylin and eosin (H&E) stained tissue slides of post-imaged sections. **(H)** QQQ MALDI-MSI showing BKI-1708 and M2 presence (yellow) in mouse small intestine and colon sections 24 h post BKI-1708 administration (n = 4). Phosphatidylcholine headgroup PC(34:1) (blue) was used for detection of tissue-containing regions. Signals were overlayed on H&E-stained tissue slide sections. BKI-1708 and M2 signals show accumulation in the mucosal layer at the luminal surface. **(I)** QQQ MALDI-MSI showing BKI-1708 and M2 presence (yellow) in the colon at 1 h versus 24 h post BKI-1708 administration (n = 4). At 24 h, the signal remains in the lumen, but is mostly absent from the intestinal wall.

To further investigate PK distribution of BKI-1708, we administered 30 mg/kg PO to Balb/c mice and at various time points over a 12 h period, extracted plasma and brain tissue to determine CNS penetration, and collected GI sections (duodenum, jejunum, ileum, and cecum/colon) to measure drug exposure at the site of infection (**Fig 5**) [27,48,79]. BKI-1708 was found to have low CNS penetration with a total brain-to-plasma drug partition coefficient (K_p,brain_) of 0.075. To determine the partition coefficient for the free fraction of drug (K_p,uu,brain_) [80], we measured the degree of brain binding via equilibrium dialysis and found BKI-1708 to be 97% bound. This resulted in a K_p,uu,brain_ of 0.053. When comparing the CNS exposures of M2 to the parent molecule, we observed an equivalent M/P AUC ratio measured in plasma in the initial PK study (3.3) (**Fig 5A and 5B**). BKI-1708 presence was highest in the duodenum, jejunum, and ileum in the first h post dose, but dropped below 1 μM after 2 h ( Fig 5C -F). Cecum/colon levels remained below 1 μM at all sampled times, however levels appeared to accumulate over time, peaking around 6–8 h post dose (**Fig 5F**). In contrast, M2 exposures were higher than the parent molecule in all GI tract sections with respective M/P AUC ratios of 4.5, 3.8, 4.5, and 2.4 for the duodenum, jejunum, ileum, and cecum/colon, respectively. For all sections except the duodenum, M2 appeared to reach peak levels >1 μM at 6 h. For the duodenum, M2 remained above 1 μM at all sampled time points (Fig 5C-F).

The intestinal distribution of BKI-1708 in CD1 mice after 30 mg/kg PO administration was visualized using Matrix-Assisted Laser Desorption Ionization mass spectrometry imaging (MALDI-MSI). Frozen sections of the intestines were taken at 1 h and 24 h time points post dose and imaged. At 1 h, BKI-1708 was abundantly detected within the lumen and epithelial layer in the small intestine (**Fig 5G**). At 24 h, approximately 7% of the BKI-1708 levels at 1 h remained in the lumen and 2% of the levels at 1 h remained within the intestinal mucosa. In the colon, a large amount of the remaining signal seemed to be within the lumen, possibly accumulated around the mucus layer (**Fig 5I**).

Lastly, we noted a disparity in measured BKI-1708 plasma levels from the PK studies in Balb/c mice compared to efficacy experiments in IFNγ-KO BL6 mice, with the latter strain exhibiting lower than expected exposures. Although compromised GI integrity due to *C. parvum* infection may account for the observed discrepancy, we considered whether there might be strain-specific differences in PK. To this end, we administered BKI-1708 to IFNγ-KO BL6 mice with equivalent PO and IV doses from the Balb/c experiments (**Table 9**). A single 15 mg/kg dose resulted in a C_max_ of 4.5 ± 1.3 μM, congruent with the results of the efficacy studies. M2 generation peaked with levels of 11.6 ± 3.5 μM at 120 min. M/P AUC ratios were calculated to be 6.9, suggesting a higher rate of biotransformation in IFNγ-KO BL6 mice. Furthermore, compared to Balb/c mice, the t_1/2_ was shorter, V_d_ and CL_p_ were higher, and %F was 100%. The IFNγ-KO BL6-specific differences in PK were significant and subsequent work on allometric scaling relied on this dataset to predict V_d_, CL_p_ and t_1/2_ in humans.

### In vivo efficacy of M2 in a *C. parvum* IFNγ-KO mouse model

**Fig 6:**
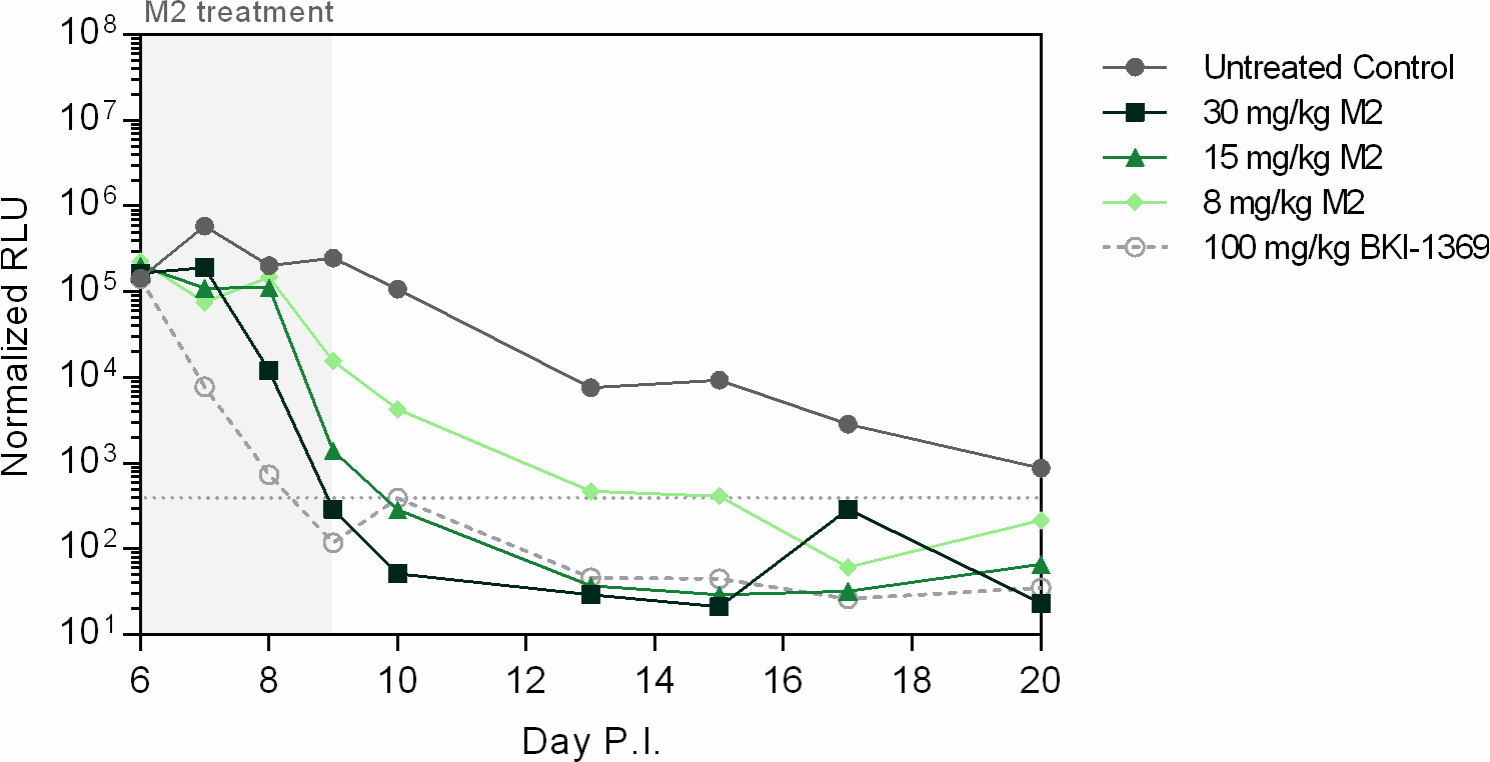
Efficacy of M2 in a mouse model of cryptosporidiosis. Mice (n = 3) are orally challenged with 10^4^ NLuc-expressing *C. parvum* oocysts on day 0 and treatment commenced on day 6. Fecal samples are collected regularly until day 20 p.i. for NLuc detection of oocyst shedding. M2 was administered PO for 3 days resulting in significant reduction in oocyst shedding for all doses compared to untreated controls. The control (light gray open circle, dashed line) used was 100 mg/kg BKI-1369 administered for 5 days, a former late-lead candidate for cryptosporidiosis [27].

In vitro potency and PK properties of M2 warranted its assessment in the *C. parvum* IFNγ-KO mouse model. We selected doses of 30, 15, and 8 mg/kg x 3 days to compare its performance against BKI-1708. Groups dosed with 30 and 15 mg/kg M2 had suppressed oocyst shedding to below the LoD by day 10 p.i. (**Fig 6**). The respective peak plasma levels were 7.7 ± 3.6 and 6.8 ± 1.2 μM (S7 Table). The 8 mg/kg group also showed significant reduction in oocysts with peak plasma exposures of 3.0 ± 1.0 μM, but did not reach the LoD until day 17 p.i. Although these results may suggest superior efficacy of M2 compared to BKI-1708, the characteristics of the *C. parvum* infection for this experiment were different from prior experiments, as evidenced by the gradual reduction of parasite numbers in untreated controls by day 20 p.i. Also, in most experiments, untreated mice exhibit rapid increases in parasite load by day 10 p.i. correlated with excessive weight loss, requiring euthanasia in this model [27,29]. These discrepancies may be attributed to older batch age of the oocysts used. However, the results demonstrate that M2 exhibits significant anti-*Cryptosporidium* activity in vivo and BKI-1708 efficacy in mice is likely mediated by both the parent and metabolite M2.

### In vivo efficacy of BKI-1708 in a newborn calf clinical model of cryptosporidiosis

**Fig 7:**
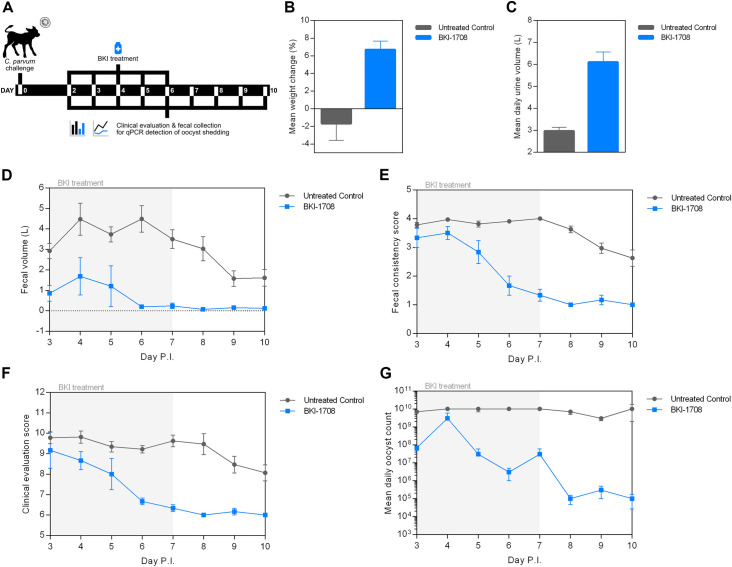
Efficacy of BKI-1708 in a calf model of cryptosporidiosis. **(A)** Experimental design of the newborn calf clinical model of cryptosporidiosis. Newborn calves (n = 3 treated; n = 16 untreated) were orally challenged with 5x10^7^ *C. parvum* oocysts on day 0 and twice daily 5 mg/kg BKI-1708 treatment commenced on day 2 for 5 days. **(B)** Mean percent change in weight from birth to the end of the experimental period, on day 10. **(C)** Mean daily urine output. **(D)** Daily fecal volumes. **(E)** Daily fecal consistency scores. Consistency scores range from 1 for normal-formed stool to 4 for severely diarrheic stool. **(F)** Daily clinical evaluation scores. Clinical evaluation scores are a summative assessment of clinical health parameters (willingness to rise, stance, appetite, attitude, and hydration status), where lower scores reflect normal/healthy calves [67]. **(G)** Mean daily oocyst counts. Fecal samples were collected daily until day 10 p.i. for qPCR detection of oocyst shedding (n = 3 technical replicates).

The efficacy of BKI-1708 was assessed in a newborn calf model of cryptosporidiosis which more closely approximates human infections with respect to clinical symptoms and diarrhea [67]. Newborn Holstein bull calves between the ages of 36 and 48 h were enrolled in the study and orally infected with 5x10^7^ *C. parvum* oocysts. Starting on day 2 p.i. a twice-daily (BID) dose of 5 mg/kg was orally administered in milk replacer for 5 days. No BKI-1708 treatment associated toxicity was observed. A significant improvement in the severity and duration of diarrhea was observed over vehicle treated controls by day 6 p.i. and remained significantly improved until day 10 p.i. as compared by total daily fecal output and fecal consistency scores (p < 0.05) (**Fig 7D and 7E**). A 2-fold increase in mean daily urine output was observed compared to untreated controls, which is consistent with a reduction in fluid loss through stool and improved hydration status. BKI-1708 treated calves showed significant improvements in clinical health scores (p < 0.05) and concluded the study with a net gain in weight (+6.7%) whereas vehicle treated calves exhibited a net loss in weight (-1.7%). Finally, daily fecal oocyst counts determined by real time-PCR (qPCR) showed that BKI-1708 treatment reduced shedding over the study period with 3.7x10^8^ mean total daily oocysts excreted compared to 1.0x10^10^ for controls, an approximately 28-fold reduction in daily parasite levels. By the end of BKI-1708 therapy, the daily output of *C. parvum* oocysts was reduced >3-log for several days compared to untreated controls (**Fig 7G**). Steady-state plasma concentrations of BKI-1708 reached peak concentrations of 9.5 ± 5.8 μM with high variability between calves. Furthermore, average peak concentrations of M2 were found to be slightly lower (7.1 ± 4.4 μM) and did not reliably surpass the levels of the parent molecule as seen in mice.

### In vivo pharmacokinetics of BKI-1708 in rats, dogs, and monkeys

Additional PK profiles were obtained for rat, dog, and monkey to help in predictions for calf and human doses (**Table 10**). IV administration revealed moderate V_d_ in dogs and high V_d_ in rats and monkeys. The CL_p_ for all species were low (<10 mL/min/kg), with dogs showing the lowest clearance. Both IV and PO routes yielded similar t_1/2_s in each species; however, the t_1/2_s for dogs were >2-fold longer than rats and monkeys. The oral bioavailability in rats and monkeys was relatively high (86% and 80%, respectively), but highest in dogs at 99%. In dogs, formation of M2 was measured after PO administration, and the M/P AUC ratio was 0.12. In monkeys, M/P AUC ratios of 0.20 and 0.24 were observed after IV and PO administrations, respectively. These results were in marked contrast to PK profiles in mice where M2 levels far surpass the exposure of the parent molecule, which is suggestive of a variance in clearance mechanisms and is congruent with the results of the metabolic profiling studies (**Fig 4**).

**Table 10:** Pharmacokinetic profile of BKI-1708 in rat, dog, and monkey.

Species	Route	Dose (mg/kg)	C_max_ (µM ± SD)	C_0_ (µM ± SD)	T_max_ (min)	t_1/2_ (min)	AUC_0-inf_ (min*µmol/L ± SD)	CL_p_ (mL/min/kg)	V_d_ (L/kg)	%F	M2 C_max_ (µM ± SD)	M2 T_max_ (min)	M2 t_1/2_ (min)	M2 AUC_0-inf_ (min*µmol/L ± SD)	M/P AUC	BKI-1708 AUC/D
Rat	PO	2	0.9 ± 0.3		258	198	479 ± 56			86	ND	ND	ND	ND	ND	240
	IV	1		0.9 ± 0.2		210	280 ± 0.40	9.2	2.8		ND	ND	ND	ND	ND	280
	PO	10	3.2 ± 0.7		260	ND	2504 ± 335*				1.5 ± 0.8	600	ND	1178 ± 565*	0.47	250
	PO	30	10.3 ± 3.9		320	ND	8902 ± 3002*				5.9 ± 2.4	660	ND	4857 ± 1917*	0.55	297
	PO	100	30.7 ± 1.6		138	330	24639 ± 1253				9.5 ± 0.7	660	720	14052 ± 2156	0.57	246
	PO	200	35.5 ± 6.6		162	438	40304 ± 11772				14.6 ± 1.4	660	570	24159 ± 5596	0.60	202
	PO	300	32.7 ± 2.5		258	438	40304 ± 2859				15.0 ± 2.7	600	582	25535 ± 5459	0.63	134
Dog	PO	1	2.2 ± 0.3		60	474	1638 ± 176			99	0.19 ± 0.0	600	ND	196 ± 12	0.12	1638
	IV	1		3.3 ± 1.1		450	1640 ± 361	1.6	1.0		ND	ND	ND	ND	ND	1640
	PO	3	5.6 ± 0.4		270	342	5125 ± 456				ND	ND	ND	ND	ND	1708
	PO	10	21.6 ± 2.5		150	168	11970 ± 760				ND	ND	ND	ND	ND	1197
	PO	30	55.5 ± 4.1		210	180	34373 ± 3498				ND	ND	ND	ND	ND	1146
Monkey	PO	1	0.5 ± 0.2		378	216	246 ± 58			80	0.1 ± 0.0	540	300	58 ± 13	0.24	246
	IV	1		1.4 ± 0.1		192	309 ± 38	8.5	2.4		0.1 ± 0.0	282	306	63 ± 9	0.20	309

PO: oral; IV: intravenous; C_max_: peak plasma concentration; C_0_: initial plasma concentration; T_max_: time at peak concentration; AUC_0-t_: area-under-curve extrapolated to infinity; CL_p_: plasma clearance; V_d_: volume of distribution; %F: oral bioavailability; M/P AUC: ratio of metabolite to parent AUCs; AUC/D: AUC divided by dose; ND: data not available.

*Values reported as AUC_0–24_: area-under-curve to 24 h time point.

Rats administered single doses of 10, 30, 100, 200, and 300 mg/kg showed increases in AUC approximately proportional to an increase in dose up to 100 mg/kg (**Table 10**). At higher doses, drug exposure appears to plateau, which may suggest saturation of passive uptake mechanisms or solubility-limited absorption. However, no dose-dependent shifts in the time at which C_max_ is reached (T_max_) were observed, which suggests that absorption is not likely to be dissolution rate-limited. The 200 mg/kg dose achieved the highest C_max_ of 35.5 ± 6.6 μM. The M/P AUC ratios were similar across all doses, but showed marginal increases at higher dose levels. Furthermore, as in dogs and monkeys, M2 exposure in rats does not appear to eclipse the levels of the parent molecule as observed in mice. Dose range finding studies were done with dogs at 3, 10, and 30 mg/kg levels (**Table 10**). Increases in AUCs were roughly dose proportional and at 30 mg/kg a C_max_ of 55.5 ± 4.1 μM was reached. Interestingly, the t_1/2_ s of the 10 and 30 mg/kg doses appeared to be about 2-fold less than that of the 3 mg/kg dose.

PK data from mouse, rat, dog, and monkey were used to inform allometric scaling for the prediction of CL_p_, V_d_, and t_1/2_ in humans. BKI-1708 is predicted to have low clearance (3.7 L/h), moderate distribution (104.4 L) and an elimination t_1/2_ of 19.6h. In vitro to in vivo extrapolation (IVIVE) from hepatocyte and microsome studies predicted slightly higher clearance rates and shorter t_1/2_s (S8 Table). A human dosing regimen was predicted based on effective BKI-1708 exposures in mice. Assuming a typical body weight of 70 kg, allometric scaling estimated a daily dose of 18 mg, while IVIVE approximated daily doses ≥45 mg (S9 Table).

### In vivo safety profile of BKI-1708

The in vivo mouse safety profile of BKI-1708 was characterized previously [29,50] with a maximum tolerated single dose of 400 mg/kg, no observed adverse effects with 200 mg/kg/day x 7 days in adult mice and 100 mg/kg/day x 7 days in freshly weaned 3-week-old mice, and no neurological issues as measured in the locomotor activity box test with 150 mg/kg/day x 5 days of dosing. A safety margin of 109.5 was estimated based on total BKI-1708 exposures from the efficacious 15 mg/kg x 3-day dose regimen and the 7-day adult mouse toxicity study. Margin estimates using exposures over a 24 h period following the first or last dose were lower (46.9) (S11 Table). To determine prenatal and postnatal safety of BKI-1708, we administered 20 mg/kg QD x 5 days PO to female Balb/c mice starting day 9 post-mating. Pregnant mice were separated on day 18 and monitored through birth on days 20–22. Fertility rates, abortions, and postnatal deaths after 14 days were compared to vehicle treated controls. BKI-1708 was safe, had no effect on pregnancy or offspring viability (**Table 11**).

**Table 11:** In vivo safety of BKI-1708 in a mouse pregnancy interference assay.

Group	Dose (mg/kg/day)	mice/group	pregnant mice	fertility rate (%)	litter size	neonatal mortality (%)	postnatal mortality (%)	Postnatal survival (%)
**control**	**vehicle**	6	3	50	16	0	0	100
**BKI-1708**	**20 QD x 5**	6	3	50	17	0	0	100

Neonatal mortality assessed 2 days post birth; postnatal viability assessed 14 days post birth.

Eight-week-old Sprague Dawley rats were administered PO doses of 30, 75, and 200 mg/kg QD for 5 days. All doses were tolerated. The 200 mg/kg/day group had ruffled hair and exhibited slight weight loss (~6%) and decreased food consumption. Histopathology findings include a reduction in thymus lymphocytes, likely attributable to stress. Plasma concentrations for the 200 mg/kg/day group reached a C_max_ of 49.4 μM for BKI-1708 and 14.7 μM for M2 (**Table 12**). Furthermore, plasma concentrations on day 5 versus day 1 were comparable or higher, suggesting minimal changes to metabolism with repeated dosing up to 5 days. Although the AUCs increased with each dose level, the increases were less than proportional. M/P AUC ratios were consistent across all doses. Safety margins of 119.6, 67.6, and 72.9 were estimated based on total, first 24 h, and last 24 h AUCs, respectively (S12 Table).

**Table 12:** In vivo toxicokinetic profile of BKI-1708 in rats and dogs.

Toxicity study	Dose (mg/kg/day)	Sex	First day BKI-1708 [M2] C_max_ (µM ± SD)	First day BKI-1708 [M2] AUC_0–24_ (min*µmol/L ± SD)	First day AUC/D	First day AUC M/P	Last day BKI-1708 [M2] C_max_ (µM ± SD)	Last day BKI-1708 [M2] AUC_0–24_ (min*µmol/L ± SD)	Last day AUC/D	Last day AUC M/P	Last day/ First day AUC	Last day/ First day M2 AUC
**Rat, 5-day**	**30**	**M**	10.9 ± 3.2 [ND]	ND	ND	ND	14.2 ± 3.3 [4.1]	14454 ± 2767 [3899]	482	0.27	ND	ND
	**75**	**M**	25.4 ± 2.3 [ND]	ND	ND	ND	22.6 ± 6.7 [7.1]	24081 ± 7149 [7217]	321	0.30	ND	ND
	**200**	**M**	21.1 ± 6.7 [ND]	ND	ND	ND	49.5 ± 27.5 [14.7]	46084 ± 21855 [14205]	230	0.31	ND	ND
**Rat, 14-day**	**30**	**M**	11.4 ± 1.9 [3.2 ± 1.4]	7050 ± 1449 [2510 ± 946]	235	0.36	9.6 ± 2.8 [2.9 ± 1.4]	7138 ± 1955 [2288 ± 852]	238	0.32	1.1	0.91
		**F**	7.5 ± 0.9 [4.6 ± 1.3]	4771 ± 923 [3920 ± 1191]	159	0.82	7.9 ± 0.2 [4.5 ± 1.7]	5287 ± 456 [3773 ± 1250]	176	0.71	1.11	0.96
	**75**	**M**	25.3 ± 9.2 [4.5 ± 1.4]	20851 ± 6965 [4818 ± 2165]	278	0.23	20.7 ± 2.4 [4.5 ± 0.5]	15744 ± 965 [4436 ± 653]	210	0.28	0.76	0.92
		**F**	17.0 ± 2.5 [8.3 ± 0.8]	11548 ± 522 [6415 ± 521]	154	0.56	13.4 ± 5.0 [5.1 ± 0.9]	7907 ± 5395 [3977 ± 2702]	105	0.5	0.68	0.62
	**200**	**M**	33.4 ± 12.3 [9.0 ± 0.3]	21246 ± 3773 [9336 ± 1388]	106	0.44	18.3 ± 2.3 [4.5 ± 1.1]	14087 ± 1845 [4994 ± 1321]	70	0.36	0.66	0.53
		**F**	25.5 ± 3.5 [12.6 ± 3.9]	13478 ± 1441 [10032 ± 2306]	67	0.74	18.2 ± 3.7 [7.5 ± 3.1]	11467 ± 898 [5865 ± 1566]	57	0.51	0.85	0.58
**Dog, 5-day**	**10**	**M**	19.7 ± 1.8 [1.8 ± 0.1]	10844 ± 1312 [1463 ± 94]	1084	0.13	11.9 ± 0.9 [0.9 ± 0.1]	4867 ± 204 [722 ± 16]	487	0.15	0.44	0.49
		**F**	11.6 ± 1.9 [1.5 ± 0.3]	5711 ± 2785 [1199 ± 278]	571	0.21	10.5 ± 1.1 [1.0 ± 0.1]	3102 ± 172 [648 ± 1.1]	310	0.21	0.54	0.54
	**30**	**M**	33.1 ± 5.9 [4.8 ± 0.6]	24791 ± 1936 [4006 ± 443]	826	0.16	25.2 ± 15.6 [2.1 ± 1.2]	10266 ± 6872 [1667 ± 1025]	342	0.16	0.41	0.42
		**F**	28.9 ± 0.7 [4.8 ± 0.5]	18631 ± 2689 [4006 ± 627]	621	0.21	18.7 ± 5.9 [2.0 ± 0.6]	6875 ± 1280 [1448 ± 396]	229	0.21	0.37	0.36
	**50**	**M**	42.2 ± 5.6 [5.8 ± 1.1]	20532 ± 6883 [4327 ± 865]	410	0.21	22.4 ± 1.0 [1.8 ± 0.1]	8989 ± 624 [1346 ± 96]	179	0.15	0.44	0.31
		**F**	33.0 ± 14.3 [5.3 ± 2.0]	16350 ± 6990 [3517 ± 778]	327	0.22	18.4 ± 4.6 [2.9 ± 0.2]	5369 ± 1344 [1294 ± 442]	107	0.24	0.33	0.37

M: male; F: female; C_max_: peak plasma concentration; AUC_0–24_: area-under-curve to 24 h time point; AUC/D: AUC divided by dose; AUC M/P: ratio of metabolite to parent AUCs; ND: Data not available.

The potential for toxicity in rats was further examined in a pre-GLP dose range study (SRI International) to find parameters for a 28-day GLP safety study. Sprague Dawley rats were administered BKI-1708 PO doses at 30, 75, and 200 mg/kg QD levels for 14 consecutive days and necropsies performed on day 15 for main study groups and day 28 for recovery groups to assess changes in hematology, serum chemistry, and histopathology. All BKI-1708 doses were tolerated. Clinical observations included discolored fur, hunched posture, and hypoactivity, however these observations were not exclusive to BKI-1708-treated animals, but also seen in control animals. No other toxicologically meaningful changes in clinical pathology were observed for any of the dose groups and no BKI-1708-related histopathology findings were reported. Toxicokinetic analysis revealed sex-specific differences, with female rats consistently showing lower overall BKI-1708 exposures and higher M/P AUC ratios compared to males (**Table 12**). Similar to the 5-day rat study, increases in AUCs with dose level were less than proportionate. However, in contrast to that study, day 14 peak plasma levels were lower compared to day 1 for the 75 and 200 mg/kg groups for both sexes. Furthermore, day 14 AUCs were 66–85% of day 1 exposures. It appears possible that prolonged exposures (beyond 5 days) with high doses of BKI-1708 in rats may induce the activity of drug-metabolizing enzymes. A safety margin of 107 was estimated based on total BKI-1708 exposures over the study period. Moreover, margin estimates using AUCs from the first and last 24 h periods were significantly lower, but remained >10 (S13 Table).

The in vivo safety profile of BKI-1708 was also characterized in dogs (SRI International). Seven-month-old Beagle dogs were dosed PO with 10, 30, and 50 mg/kg for 5 days. All doses were well tolerated and all animals survived the study period. Clinical pathology evaluations at pre-dose and day 6 showed no differences in hematology and clinical chemistry, and all parameters fell within normal ranges. Toxicokinetic analysis revealed sex-specific differences similar to the 14-day rat study where female animals showed lower overall BKI-1708 exposures compared to males and the M/P AUC ratios were marginally higher (**Table 12**). The increases in AUCs did not scale proportionately to dose level and a significant reduction in exposures (>2-fold) were observed on day 5 versus day 1 for all dose groups. These results suggest repeated BKI-1708 dosing in dogs may have led to induction of metabolizing enzymes within the 5-day study. To further evaluate potential induction of CYP enzymes, additional in vitro studies were conducted in dog hepatocytes. BKI-1708 and M2 were not found to be inducers of CYP3A12 (the canine CYP3A ortholog). However, BKI-1708 and M2 were found to be strong inducers of CYP1A1 and CYP1A2, which may account for the >50% reduction in exposures after 5 days of dosing, as CYP phenotyping studies suggest approximately 10% and 47% contribution of CYP1A2 to BKI-1708 and M2 metabolism, respectively (**Tables 5** and **7**). Safety margins of 90.2 and 80.1 were estimated based on total and first 24 h AUCs, respectively. Margin estimates using AUCs from the last 24 h were greatly reduced (28.1) (S14 Table).

The cardiovascular (CV) safety profile of BKI-1708 in rats and dogs was summarized previously [49]. Both BKI-1708 and M2 exhibited a positive hERG signal in vitro with IC_50_s <20 μM (**Table 3**), but hERG risk is driven by the free-fraction of drug in blood, and this risk is mitigated by the high PPB of both BKI-1708 and M2 in humans (96%). Follow up evaluation in the QTiSA cardiomyocyte screen did not show any significant effects up to 30 μM. The QTiSA assay only measures direct inotropic effects on myocytes; therefore any indirect or compensatory changes that may be observed in vivo are not detected in this system. Hence, in vivo experiments are critical for accurate assessment of CV liability. CV safety was investigated in anesthetized rats and dogs using three escalating 30 min IV infusions with continual monitoring of CV parameters. In rats, IV infusion of BKI-1708 up to 60 μM did not produce significant changes in mean arterial pressure (MAP), heart rate (HR), or cardiac contractility (**Table 13**). In dogs, IV infusions up to 95 μM had no appreciable effect on MAP, HR, cardiac contractility, systemic vascular resistance (SVR), cardiac output (CO), QT interval, ventricular depolarization time (QRS), or atrioventricular conduction time (PR) (**Table 13**).

**Table 13:** In vivo cardiovascular safety profile of BKI-1708 in rats and dogs.

Species	IV infusion dose (mg/kg)	Timepoint (min)	Plasma concentration (µM ± SEM)	MAP (% change)	HR (% change)	dP/dt@50 (% change)	SVR (% change)	CO (% change)	QTcV (ms)	QRS (ms)	PR (ms)
**Rats**	**3**	**30**	4.5 ± 0.4	-4	-2	-10					
	**10**	**60**	24 ± 3.2	3	-4	-8					
	**30**	**90**	60 ± 6.8	6	-6	0					
**Dogs**	**2.1**	**15**	4.1 ± 0.2	1	5	3	2	-2	-2	0	-3
		**30**	6.6 ± 0.3	2	6	3	-6	7	0	0	0
	**7**	**45**	20.3 ± 1.7	2	4	3	-6	6	-4	-1	0
		**60**	28.5 ± 1.1	2	5	4	-1	2	-3	-1	-1
	**21**	**75**	67.6 ± 2.9	3	6	5	0	-1	-3	3	0
		**90**	94.7 ± 3.0	3	5	4	-3	1	-1	1	-2
		**105**	56.7 ± 1.3	2	3	7	-13	9	-1	3	1
		**120**	45.2 ± 3.9	1	2	7	-12	9	-1	2	-1
		**135**	41.1 ± 2.3	2	2	7	-6	8	-1	1	2
		**150**	35.8 ± 1.6	0	-3	4	-5	7	0	0	4

MAP: mean arterial pressure; HR: heart rate; dP/dt@50: cardiac contractility; SVR: systemic vascular resistance; CO: cardiac output; QTcV: QT interval corrected using Van der Water formula; QRS: ventricular depolarization time; PR: AV conduction time. A change of >15% for MAP, HR, SVR, and CO is considered biologically relevant. A change of >20% for dP/dt@50 is considered biologically relevant. A > 10 ms increase in QTcV is cause for concern (ICH E14 criteria).

## Discussion

BKI-1708 is a kinase inhibitor that targets CDPK1 in *Cryptosporidium* spp. In *Toxoplasma*, CDPK1 modulates the calcium-dependent release of secretory proteins from apical organelles that are essential for motility, invasion, and egress [42]. Similar to *Toxoplasma*, recent studies in *C. parvum* elucidated the essentiality of CDPK1 for parasite survival and identified its expression during the stages of merogeny, a period of rapid asexual proliferation where egress, motility, and invasion are critical [45]. Conventional in vitro *C. parvum* culture techniques are restricted to the initial 72 h of the parasite life cycle, a period that encompasses the cycles of merogeny [81], and thus sufficient for capturing the effects of BKI treatment. Indeed, BKI-1708 potency was demonstrated in vitro with sub-micromolar EC_50_s against several zoonotic *C. parvum* isolates as well as the anthroponotic *C. hominis*. Furthermore, electron micrographs of *C. parvum* infected monolayers after BKI-1708 treatment offer visual corroboration of its effectiveness to arrest parasite growth during this period of development (**Fig 2**).

Various in vitro and in vivo assessments performed herein show BKI-1708 has characteristics of a safe preclinical candidate. As ATP-competitive protein kinase inhibitors, the principal concern with BKIs is the potential for off-target inhibition of host kinases. Because kinases phosphorylate molecules that govern important cellular processes such as proliferation, differentiation, and apoptosis, BKI interference may result in disruptions of essential pathways and lead to undesirable toxicological effects [49]. Despite the selectivity conferred by the bulky substituent (the “bump”), some mammalian kinases have topologies beyond the gatekeeper that may accommodate binding of BKIs[22]. Kinase safety studies performed herein indicate that BKI-1708 has low promiscuity for host kinases, with only MEK1, RIPK2, and PKD3 being potentially inhibited at sub-micromolar levels. The cellular levels of inhibition for these kinases are likely to be much higher than in vitro IC_50_s because of the ATP-competitive nature of BKI-1708 inhibition, and the high concentrations of intracellular ATP in vivo (mM) vs. the ATP concentrations of in vitro assays (µM). MEK1 and PKD3 have been reported as potential therapeutic targets for BRAF-mutant carcinomas [82] and estrogen receptor-negative breast cancer [83], respectively. RIPK2 is a therapeutic target for autoimmune and inflammatory diseases such as inflammatory bowel disease (IBD) and Crohn’s disease [84,85] and is an emerging therapeutic target in metastatic castration-resistant prostate cancer [86]. Developmentally normal PKD3 and RIPK2-deficient mouse lines have been generated, which demonstrate the relative dispensability of these kinases [87,88]. However, knockout of MEK1 was reported to be lethal to mouse embryos in mid-gestation due to reduced vascularization of the placenta [89]. Thus, sustained inhibition of MEK1 may pose a risk for treatment during pregnancy. However, we show here that four MEK1 proliferation-dependent cell lines were not appreciably inhibited by 3 µM concentrations of BKI-1708, but were inhibited by 7–60 nM of MEK1 inhibitor Trametinib (S1 Fig). In addition, we demonstrated the safety of BKI-1708 on zebrafish embryo development at concentrations up to 2 µM. As fertilized eggs are directly exposed to drug dissolved in aqueous medium, they are highly sensitive to drug-induced effects and serve as a screen for teratogenesis. In mammals, a considerable proportion of drug may be sequestered with plasma proteins and unavailable to engage liability targets. Indeed, BKI-1708 was found to be highly protein bound (>95%) in plasma of pre-clinical species and humans, indicating an additional buffer against potential toxicity. This also suggests MEK1 cellular inhibition is unlikely to occur during therapeutic exposures of BKI-1708. Furthermore, BKI-1708 showed excellent safety when administered to pregnant mice, exhibiting no effects on fertility or pre-natal and post-natal survival rates. There are no cardiovascular risks of BKI-1708 therapy that could be demonstrated in preclinical models. While in vitro screens indicated hERG channel inhibition at 13 and 6 µM levels (BKI-1708 and M2, respectively), these risks are greatly reduced by the > 95% PPB of BKI-1708 and M2, keeping the f_u,p_ below 1 µM during efficacious dosing. In vitro QT assessment in rabbit cardiomyocytes showed no inotropic effects with BKI-1708 exposure. Moreover, excellent in vivo cardiovascular safety was demonstrated in rats and dogs with IV infusions of BKI-1708 at levels >10-fold higher than therapeutic exposures in mice.

M2 appeared to be the primary metabolite species in mouse and human hepatocytes, while in rats, dogs, and monkeys the O-glucuronidated product, M4, appeared predominate. This divergence suggests that the metabolic pathways in these pre-clinical species differ. However the clearance rate of BKI-1708 and M2 was more similar in rats, dogs, and human LMs and hepatocytes than in mice. Thus it is difficult to predict whether the M2 predominance observed in mice after oral dosing of BKI-1708 will be predictive of what will be observed in humans. Efforts to identify the metabolic enzymes responsible for the formation of M2 were inconclusive. CYP phenotyping studies suggested CYP3A4 to be the main contributor to BKI-1708 metabolism. However, hepatocyte incubations with a CYP3A4 specific inhibitor only resulted in a modest reduction in clearance (<2-fold), while incubations with a pan-CYP inhibitor resulted in >10-fold lower clearance. Thus, there was a discrepancy between CYP phenotyping vs. the inhibitor studies. The results suggest CYP involvement in BKI-1708 metabolism, but the primary CYP isoform responsible for metabolism and M2 generation may not have been captured in the phenotyping panel.

In mice, M2 was rapidly generated, reaching peak plasma levels 2 h after BKI-1708 administration and eclipses the systemic exposures of the parent molecule by up to 7-fold. M2 levels were also elevated above BKI-1708 in the CNS and had extended presence in all sections of the GI tract. MALDI-MSI revealed that BKI-1708 and M2 appeared to accumulate in the intestinal lumen and within the mucosal layer, which is the primary site of infection where parasites egress from their parasitophorous vacuoles on the epithelial surface and enter the luminal space to invade other enterocytes. The M2 metabolite retains sub-micromolar activity against *Cryptosporidium* spp. Moreover, oral administration of M2 was effective in suppressing oocyst levels in mice at equivalent dosage levels of BKI-1708. These findings suggest that M2 contributes to the efficacy of BKI-1708 in mice. If this DMPK profile of high M2 exposure is recapitulated in humans as predicted by in vitro hepatocyte metabolism studies, the auxiliary exposures of M2 may be important for treating biliary and pulmonary infections in exceptional cases of cryptosporidiosis [90–92]. While these results suggest M2 could be a preclinical candidate, rather than BKI-1708, several factors negate further consideration of its development. First, from a safety standpoint, M2 showed greater activity against the hERG channel in vitro compared to BKI-1708 although this is mitigated by high PPB. The peak f_u,p_ levels are likely to be lower when M2 appears as a metabolite, rather than being directly absorbed in the GI tract. Next, more cytotoxicity was observed for M2 than BKI-1708 in lymphocyte and hepatocyte cell lines and could exacerbate toxicity when only M2 is delivered. Finally, there is some uncertainty regarding which form of M2 drives potential toxicity or efficacy. M2 is a product of dehydrogenation of the terminal alcohol of BKI-1708; the resulting aldehyde can isomerize to form a stable cyclized hemiaminal of identical mass, which can interconvert. We were unable to synthesize the pure aldehyde for further studies but a synthetic method for generating the hemiaminal was identified, albeit with a low overall yield. While the hemiaminal was used for all in vitro and in vivo studies described herein, the question remains whether the hemiaminal is the active M2 isomer or whether isomerization to the aldehyde is required for its activity. Efforts to distinguish between the two isomers using standard C18 reverse-phase or normal-phase mass spectrometry have proven difficult, and attempts to resolve the two products in complex biological matrices to identify the predominant form in vivo were unsuccessful. The possible safety issues coupled with the difficulties in chemical synthesis, the propensity for interconversion, and ambiguity of the active isomer make M2 a less attractive compound to progress as a drug candidate. Yet, in the context of BKI-1708 treatment, the complementary activity of M2 appears valuable and offers the prospect of a dually active drug that extends therapeutic potential.

Potent in vivo efficacy of BKI-1708 was previously demonstrated in a mouse model of *C. parvum* infection with a 5-day regimen of 8 mg/kg QD [29]. Herein, we show that an abbreviated oral dose regimen of 15 mg/kg for 3 days also results in complete suppression of oocyst shedding in mice. Moreover, a significant reduction of oocyst shedding was achieved with a single dose as low as 30 mg/kg but with a reduced rate of parasite clearance. It is unclear whether this would be adequate for parasitological cure or whether rapid clearance of parasites offers the best chance for therapeutic success in diarrhea models of cryptosporidiosis. Live imaging studies have shown that the *C. parvum* life cycle follows a strict timetable that involves 3 generations of asexual merogeny, with each cycle lasting approximately 12 h, followed by a single generation of sexual reproduction [47]. This rigid developmental program suggests that BKI-1708 has a trio of opportunities during each life cycle to exert its effect. Asynchronous infection and requirements for multiple exposures during merogony may explain why 3-day therapy is able to eliminate oocyst production in the *C. parvum* mouse model we used (**Fig 3**).

Although mice provide an excellent platform for initial in vivo testing of drug efficacy against *Cryptosporidium*, one limitation of the model is that fecal elimination in rodents is different compared to other mammals, with decreased fecal dry matter often manifesting only as moist pellets of solid stool [93]. This is distinct from the profuse watery feces characteristic of cryptosporidiosis diarrhea in humans and calves. Normally, the human stomach produces about 2.5 L of fluid per day, a bulk of which is absorbed by the small intestine and colon, leaving approximately 100 mL that is expelled in stool [94,95]. In *Cryptosporidium* infections, the absorptive capacity of the GI tract is severely compromised, leading to increased GI secretions, excessive fluid loss, and decreased intestinal transit times. These factors may significantly impact drug absorption and GI residence times and limit efficacy as drugs are quickly washed out. Therefore, efficacy assessment in a second animal model that more closely resembles human cryptosporidiosis diarrhea is critical. To this end, a *C. parvum* newborn calf model was used to substantiate the efficacy of BKI-1708. Cryptosporidiosis is endemic in cattle and a leading cause of morbidity and mortality due to diarrhea in pre-weaned calves [96,97], with symptoms that mirror human infections including watery diarrhea, dehydration, and poor nutrient absorption. BKI-1708 administered at 5 mg/kg BID for five days resulted in rapid improvements in diarrhea and clinical symptoms, and produced a > 3 log reduction in daily parasite levels at the end of therapy. Treated calves also showed significant weight gain over the study period, whereas control calves lost weight. These results demonstrate that BKI-1708 is efficacious in a model that closely approximates the clinical manifestations of disease in humans. Work is ongoing to investigate other effective dose regimens for veterinary treatment of cryptosporidiosis in calves.

BKI-1708 has good systemic exposure, low clearance, and high oral bioavailability in rats, dogs, and monkeys. Allometric scaling predicts low clearance, moderate distribution, and a t_1/2_ of approximately 19.6 h in humans. Furthermore, a daily dose in the range of 0.26-1.1 mg/kg is expected to maintain efficacious exposures. Unlike in mice, M2 levels remained well below BKI-1708 exposures across rats, dogs, and monkeys, with dogs showing the lowest M/P AUC ratios. This finding is in congruence with the metabolite profiling in cross-species hepatocytes. BKI-1708 showed excellent safety margins in repeated-dose studies in rats and dogs. However, plasma exposures did not increase with daily dosing in either species. This may be suggestive of induction of clearance mechanisms with prolonged BKI-1708 treatment. Indeed, RNA expression data show that CYP1A1 and CYP1A2 are strongly induced in dogs. As an inducer of CYP1A isoforms, BKI-1708 may likely be an Aryl hydrocarbon nuclear receptor (AhR) agonist. AhR is a ligand-dependent transcription factor that regulates the expression of several phase I and phase II metabolizing enzymes including the CYP1A isoforms and some UDP-Glucuronosyltransferases (UGTs) [98,99]. Moreover, as the major metabolite species in rats and dogs is the O-glucuronide, M4, repeated dosing may also induce the expression of UGTs through AhR activation to result in increased biliary elimination and reduced systemic availability of BKI-1708 in these animals. Nevertheless, the target therapeutic regimen for clinical treatment of cryptosporidiosis is projected to be short, likely less than 5 days in duration, and at these timeframes, the effects of induction are expected to be minimal.

The 5-aminopyrazole-4-carboxamide inhibitor BKI-1708 represents an exciting new class of anti-*Cryptosporidium* agent that can meet the demands of a TPP for an ideal cryptosporidiosis drug. BKI-1708 is potent against both *C. parvum* and *C. hominis* isolates relevant to human disease. It has excellent efficacy in an immune-deficient mouse model at low doses and leads to rapid resolution of diarrhea and improvements in clinical symptoms in a newborn calf model of cryptosporidiosis diarrhea. It shows remarkable cardiovascular, CNS, and prenatal and postnatal safety in animal models. Finally, its metabolism results in an active compound that has high systemic and GI presence in mice, and likely in humans, which contributes to its therapeutic value. These results strongly support advancement of BKI-1708 to clinical trials for the evaluation of safety and efficacy in treating cryptosporidiosis in humans.

## Supporting information

S1 File Methods [100–102]. (PDF)

S1 Fig Cellular proliferation assays to screen for MEK inhibition. (PDF)

S2 Fig Cerep cellular and nuclear receptor functional assay: agonist and antagonist effect of BKI-1708 on PPARy. (PDF)

S3 Fig Cerep binding assay: agonist effect of BKI-1708 metabolite, M2 on AT_2_. (PDF)

S4 Fig Cerep binding assay: antagonist effect of BKI-1708 metabolite, M2 on Cl^-^ channel (GABA-gated). (PDF)

S5 Fig Cerep cellular and nuclear receptor functional assay: agonist and antagonist effect of BKI-1708 metabolite, M2 on A_3_. (PDF)

S6 Fig Cerep cellular and nuclear receptor functional assay: agonist and antagonist effect of BKI-1708 metabolite, M2 on PPARy. (PDF)

S7 Fig Cerep cellular and nuclear receptor functional assay: agonist and antagonist effect of BKI-1708 metabolite, M2 on 5-HT_2B_. (PDF)

S8 Fig M2 synthesis scheme 1. (PDF)

S1 Table Efficacy of BKI-1708 in the IFNγ-KO mouse model of cryptosporidiosis. BKI-1708 [M2] plasma exposures before and after final administration.
(PDF)

S2 Table BKI-1708 and M2 metabolite activity against the bioprofiling panel of 20 common liability targets (37 functional assays). (PDF)

S3 Table BKI-1708 activity against the Cerep panel of 71 common liability targets: Binding assays. (PDF)

S4 Table BKI-1708 activity against the Cerep panel of 71 common liability targets: enzyme and Uptake assays. (PDF)

S5 Table BKI-1708 metabolite, M2 activity against the Cerep panel of 71 common liability targets: binding assays. (PDF)

S6 Table BKI-1708 metabolite, M2 activity against the Cerep panel of 71 common liability targets: enzyme and Uptake assays. (PDF)

S7 Table Efficacy of M2 in the IFNγ-KO mouse model of cryptosporidiosis. M2 plasma exposures before and after final administration.
(PDF)

S8 Table Predicted human BKI-1708 PK parameters and half-life by allometric scaling or in vitro to in vivo extrapolation. (PDF)

S9 Table Predicted human BKI-1708 dosing in 70 kg human based on effective mouse BKI-1708 exposure. (PDF)

S10 Table List of toxicology studies in mice, rats, and dogs. (PDF)

S11 Table Safety margin for BKI-1708 with 5–7 day mouse study. (PDF)

S12 Table Safety margin for BKI-1708 with 5-day rat study. (PDF)

S13 Table Safety margin for BKI-1708 with 14-day rat study. (PDF)

S14 Table Safety margin for BKI-1708 with 5-day dog study. (PDF)

S1 Data Calibr *Cryptosporidium* panel screens. (XLSX)

S2 Data *C. parvum* IFNγ-KO mouse efficacy fecal collection weights and raw RLU measurements. (XLSX)

S3 Data Mouse plasma exposures and PK parameters. (XLSX)

S4 Data Mouse brain and GI exposures. (XLSX)

S5 Data Newborn calf model of cryptosporidiosis clinical assessments and oocyst counts. (XLSX)
