# Supplementary material for: Anti-Cryptosporidium efficacy of BKI-1708, an inhibitor of Cryptosporidium calcium-dependent protein kinase 1
Source: PLoS Negl Trop Dis. 2025 Jul 30;19(7):e0013263. doi: 10.1371/journal.pntd.0013263 (PMC12310023; doi:10.1371/journal.pntd.0013263)
Supplement: S1 File — (PDF) [file pntd.0013263.s001.pdf]

Supplemental Methods for

Anti-*Cryptosporidium* efficacy of BKI-1708, an inhibitor of *Cryptosporidium* calcium-dependent protein kinase 1

Ryan Choi, Matthew A. Hulverson, Deborah A. Schaefer, Dana P. Betzer, Michael W. Riggs, Wenlin Huang, Vicky Sun, Grant R. Whitman, Molly C. McCloskey, Kennan Marsh, Wayne R. Buck, David S. Wagner, Junhai Yang, Andrew P. Bowman, Rita Ciurlionis, Jubilee Ajiboye, Andrew Hemphill, Dilep K. Sigalapalli, Samuel L.M. Arnold, Lynn K. Barrett, Kayode K. Ojo, Erkang Fan, Wesley C. Van Voorhis*

*wesley@uw.edu

Chemical synthesis of M2 metabolite: 2-(6-cyclopropoxynaphthalen-2-yl)-5-hydroxy-6,6-dimethyl-4,5,6,7-tetrahydropyrazolo[1,5-a]pyrimidine-3-carboxamide from BKI-1708

Compound BKI-1708 (0.25 mmol) was dissolved in 5 mL of dry dichloromethane, and the solution was cooled to 0°C. Dess-Martin periodinane (0.50 mmol) was slowly added to the solution under nitrogen atmosphere and the reaction was allowed to stir at 0°C for 20 min (**S8A Fig**), resulting in complete consumption of the starting material determined by TLC. The reaction mixture was quenched with a saturated sodium thiosulphate solution and washed with an aqueous $NaHCO_3$ solution. The reaction mixture was extracted with dichloromethane (2 × 25 mL), and the solvent was evaporated using a rotary evaporator. The residue was then purified by column chromatography. Initially, a silica gel column (eluent EtOAc/DCM, 50/50) was used to purify the crude reaction mixture. Then a second purification was performed with a neutral alumina column. THF was passed through the column to remove any color impurities. Further, MeOH/dichloromethane (4/96) was utilized as an eluent to obtain the pure product (M2, yield = 15%). The separated product was precipitated with DCM and hexane, then dried and analyzed immediately using LC-MS and NMR (**S8B Fig**). *2-(6-Cyclopropoxynaphthalen-2-yl)-5-hydroxy-6,6-dimethyl-4,5,6,7-tetrahydropyrazolo[1,5-a]pyrimidine-3-carboxamide* (M2): Gray solid, ^{1}H NMR (500 MHz, DMSO-d_6): δ 8.01 (d, *J* = 1.8 Hz, 1H), 7.90 (dd, *J* = 9.0, 2.3 Hz, 2H), 7.63 – 7.58 (m, 2H), 7.21 (dd, *J* = 8.9, 2.5 Hz, 1H), 7.14 (d, *J* = 3.5 Hz, 1H), 5.87 (d, *J* = 4.0 Hz, 1H), 4.46 (t, *J* = 3.8 Hz, 1H), 3.99 (tt, *J* = 6.0, 2.9 Hz, 1H), 3.80 (d, *J* = 12.0 Hz, 1H), 3.70 (d, *J* = 12.1 Hz, 1H), 1.09 (s, 3H), 0.97 (s, 3H), 0.88 (m, 2H), 0.74 (m, 2H). MS (m/z): 393.2 (M+H).

Analytical quantification of BKI-1708 and M2 plasma levels

All LC-MS/MS analytes were measured with an Acquity ultra performance liquid chromatography (UPLC) system in tandem with a Xevo TQ-S micro mass spectrometer (Waters, Milford, MA, USA). Stock samples were prepared for each test compound or internal standard (IS) in DMSO at a concentration of 20 mM. Stock solution for propranolol was stored at RT away from light. Stock solutions for BKI-1517, -1708, and M2 were stored frozen at -20ºC. To prepare standard curves (SC) in matrix, stock solutions were diluted in DMSO to obtain working concentrations of 200 to 0.09 µM, and 18 µL of blank matrix (i.e. mouse plasma, homogenized tissue, etc.) was spiked with 2 µL of DMSO working solutions. Five µL of standard curve sample, experimental sample, or QC sample was pipetted into each well of a 96-well cone bottom plate, followed by 95 µL of acetonitrile mix containing IS (80% ACN / 20% Water / 200 nM IS) in each well. Plates were sealed with a 96-well silicone cap mat, shaken at 10000-12000 rpm for 30-60 min on a microplate shaker, centrifuged at 4000 rpm for 10 min, and 95 µL of supernatant were transferred to a new plate.

UPLC conditions for BKI-1708, M2, and IS BKI-1517 and propranolol were as follows: Column: Agilent ZORBAX Eclipse Plus C18 Column Rapid Resolution HD (2.1 x 50 mm, 1.8 µm) (Agilent Technologies, Santa Clara, CA); Column temperature: 40ºC; Sample temperature: 10ºC; Injection volume: 5 µL. Mobile phase A: 0.1% formic acid in LCMS grade water; mobile phase B: 0.1% formic acid in LCMS grade acetonitrile (ACN); and the gradients are as follows:

Time	Flow rate (mL/min)	Phase A %	Phase B %
**0**	0.25	95	5
**1**	0.25	95	5
**5**	0.25	5	95
**6.5**	0.25	5	95
**6.6**	0.25	95	5
**8**	0.25	95	5

The Xevo TQ-S micro MS was used in positive mode with a source temperature of 150°C and source gas flow rate of 650 L/hr. The selected m/z transitions and compound dependent parameters are as follows:

Compound	Parent m/z	Fragment m/z	Cone (v)	Collision (v)
**Propranolol (IS)**	260.1595	127.335	46	44
**BKI–1517 (IS)**	354.1766	281.0913	76	26
**BKI-1708**	395.1681	292.1257	28	30
**M2**	393.1681	320.1012	28	28

Peaks were analyzed using software MassLynx v4.2 (Waters, Milford, MA, USA). Standard curves were constructed for each LC-MS/MS run by plotting the compound/IS peak area ratio versus the expected concentration of each standard sample. Curves consisted of 12 concentrations of compound from 20 to 0.009 µM. Quality control (QC) samples of 20, 2, and 0.2 µM were prepared in a similar method to the SC samples. For each LC-MS/MS run, the trend line for the SC was required to have an R^2 value >0.98 and all points were required to be within ±15% of expected concentrations. Two of the three QC samples are required to be within ±15% of expected concentrations for each LC-MS/MS run. QC samples of BKI-1708 resulted in percent accuracy ranging from 100.4 – 114.4% with reproducibility (%CV) ranging from 12.5-23.3%. Concentration of each sample was calculated using the compound/IS peak area ratio in the equation for the SC trend line from the associated run.

SEM and TEM of *C. parvum* infected HCT-8 cells

HCT-8 cells grown on glass coverslips for SEM or in 6 well plates for TEM were infected with excysted *C. parvum* oocysts (5×10^6 and 5×10^7, respectively) at 37°C and 5% CO_2. After 3 h, BKI-1708 (2.5 µM) or the corresponding concentration of DMSO was added, and cultures underwent continuous treatment for 45 h. Specimens were fixed as previously described [100, 101]. For TEM, primary fixation was carried out in 100 mM sodium cacodylate pH 7.3/2% glutaraldehyde for 4 h at ambient temperature, and adherent cells were removed with a rubber cell scraper. Following centrifugation, post-fixation was carried out in 2% osmium tetroxide in cacodylate buffer for 2 h. After several washes in distilled water, specimens were dehydrated in ethanol, and embedded in Epon 812 epoxy resin. Following polymerization of the resin at 60°C overnight, ultrathin (80 nm) sections were cut using an ultramicrotome (Reichert and Jung, Vienna, Austria). Sections were transferred onto formvar-carbon-coated 200 mesh nickel grids (Plano GmbH, Marburg, Germany), stained with Uranyless and lead citrate (both from Electron Microscopy Sciences, Hatfield PA, USA), and imaging was performed on a FEI Morgagni TEM equipped with a Morada digital camera system (12 Megapixel) operating at 80 kV. For SEM, all fixation and dehydration steps were done on glass coverslips. After a final dehydration step in 100% ethanol, samples were twice immersed in hexamethyl-disilazene and were air-dried. Specimen were sputter coated with gold and inspected on a Zeiss Gemini 450 SEM operating at 5 kV.

Polymorph screen (WuXi AppTec & STA Pharmaceutical R&D Co., Ltd.)

Solid state stability studies were conducted using free base amorphous material. Bulk stability was assessed in an open container at 40°C/75% relative humidity and at 60°C in an air tight container for up to 2 weeks. Hygroscopicity was

evaluated by dynamic vapor sorption (SPSadv-1μ) test at 25°C. Polymorphic behaviors were investigated by equilibrium, slow cooling, slow evaporation, and anti-solvent addition experiments. Samples were analyzed by X-ray powder diffraction (Bruker D8 Advance, Bruker, Billerica, MA), differential scanning calorimetry (TA Discovery 2500, TA Instruments, New Castle, DE), thermogravimetric analysis (Discovery TGA 5500, TA Instruments, New Castle, DE), ^{1}H-NMR (Bruker Avance-AV 400M, Bruker, Billerica, MA), and Karl Fischer titration (Mettler Toledo Coulometric KF Titrator C30).

CYP Induction Assay

Cryopreserved beagle dog primary hepatocytes (Thermo Fischer Scientific, Waltham, MA) were plated at a seeding density of 0.5 x 10^6 cells per well on 12-well, collagen-type I coated plates (BD Biosciences, Bedford, MA) in hepatocyte plating medium - Williams E media with 5% fetal bovine serum, Supplement A, and dexamethasone (Thermo Fischer Scientific, Waltham, MA). Cells were allowed to attach for 4 hours in a 37°C humidified incubator with a 5% CO_2 atmosphere. Plating media was aspirated and replaced with 1ml of 0.25 mg/ml Matrigel (Corning, Bedford, MA) in cold hepatocyte incubation media (Williams E media with Supplement B and dexamethasone). After 24 hours, Matrigel overlay was aspirated and cells were dosed for 48 hours with vehicle control (0.1% DMSO (v/v)), test item at 30 μM and 100 μM and positive controls, rifampin 20 μM, phenobarbital 1000 μM, 3-methylcholanthrene 2 μM, in fresh incubation media. Following treatment, media was aspirated, and cells were lysed in 1 mL of Qiazol (Qiagen, Germantown, MD). Total RNA was isolated according to manufacturer's protocol, concentration was determined by Qubit (Thermo Fischer Scientific, Waltham, MA) and RNA integrity was evaluated on Tapestation Bioanalyzer (Agilent Technologies, Foster City, CA). RNA samples were diluted with RNase-free water to 20 ng/μL. 2.5 μL (50 ng) of diluted total RNA was reacted with 17.5 μL master mix containing iTaq Universal Probes One-Step kit (Bio-Rad, Hercules, CA) reaction mix, iScript reverse transcriptase, inventoried TaqManGene Expression Assay for canine CYP3A12, CYP2B6, CYP1A1, CYP1A2 or human 18S (Thermo Fischer Scientific, Waltham, MA) and water for a 20 μL total reaction volume. Real time PCR was performed on ViiA7 instrument with a 384 well block (Thermo Fischer Scientific, Waltham, MA) using kit manufacturer's recommended cycling parameters. Fold change was calculated by deltadeltaCt method using 18S as housekeeping gene for normalization.

Repeat dose exploratory toxicology study in rat (AbbVie, Inc.)

7-8 week old male IGS Crl:CD(SD) rats were housed 3 per cage in polycarbonate individually ventilated hanging solid bottom cages with wood chip bedding, certified wood block and other enrichment, Harlan 2014 rodent chow ad libitum, and automatic water (non-acidified) ad libitum on a 6 am-on/6 pm-off light/dark cycle at 71 ± 6°F, 30-70% RH. Rats were randomized into treatment groups of 3. Body weight, food consumption, clinical observations, dosing, and gross observations were electronically recorded (Prestima, Xybion, Princeton, NJ). For drug level, blood collected into EDTA microtubes during < 3 min restraint from the tail vein was centrifuged 1000 x g for 8 minutes and plasma was frozen within 2 hours to -20°C or colder until analysis. Rats were fasted overnight with water ad libitum prior to necropsy. Rats were anesthetized with isoflurane (4%) prior to exsanguination and necropsy. Hematology and serum chemistry were analyzed on Advia 2120i (Siemens) and Architect (Abbott) instruments, respectively. Gross observations and histopathological evaluation by a board certified veterinary pathologist of H&E stained slides from neutral buffered formalin-fixed tissue prepared by standard paraffin microtomy on liver, spleen, thymus, kidney, heart, lung, gastrointestinal tract, pancreas, and sternum (bone marrow).

MALDI mass spectrometry imaging (AbbVie, Inc.)

Male CD1 mice (n=4), ~8 weeks old, were dosed PO with 30 mg/kg BKI-1708 via gavage. At 1 h and 24 h post dose, necropsies were performed, and GI sections were extracted, embedded in gelatin which had been heat treated at 85°C for 3 days to reduce viscosity, and frozen in liquid nitrogen. Tissue blocks were cryosectioned at 16 µm thickness using a cryostat microtome and transferred to indium tin oxide-treated conductive microscope slides. Sections were coated with MALDI matrix (10 mg/mL 2,6-dihydroxyacetophenone, 15 µL/mL trifluoroacetic acid in acetonitrile:water (9:1 v/v)) in 4 passes using a HTX TM-Sprayer (HTX Technologies, LLC, Chapel Hill, NC, USA) with a 75°C nozzle, and parameters of 2 mm/track, 1100 mm/min, and 0.1 mL/min flow rate. Mass spectrometry imaging was performed using Bruker Trapped Ion Mobility Spectrometry (TIMS) time-of-flight instrument timsTOF fleX MALDI-2 (Bruker, Billerica, MA, USA) and Sciex 6500+ (Sciex, Framingham, MA, USA) triple-quadrupole (QqQ) mass spectrometer with an atmospheric-pressure MALDI source from MassTech, Inc, the AP/MALDI-UHR. (Columbia, MD, USA). timsTOF MSI sessions were carried out on a Bruker timsTOF fleX mass spectrometer in positive ion mode. The laser was set for 30 shots/pixel with laser frequency at 1000 Hz with 10 ms trigger delay. Stage and smart beam profile (spatial resolution) was 30 µm, with trapped ion mobility active and MALDI-2 laser on. Data was processed with Bruker SCiLs Lab MVS 2024a Pro. QqQ MSI sessions were carried out on a Sciex 6500+ mass spectrometer: Transitions monitored were 184.15 = Phosphatidylcholine headgroup of PC(34:1), 292.25 = Major Fragment of M2, 292.30 = Secondary fragment of BKI-1708, 378.20 = Major Fragment of BKI-1708. Summed (blue and yellow) images were generated for each intestine sample, Blue = PC(34:1), Yellow = All transitions for BKI-1708 and M2. Ion images were created with 99.5% hotspot removal. Samples were imaged at 20 µm pixel resolution. AP/MALDI settings were laser power at 6%, repetition rate of 5000 Hz. Transitions were accumulated for each, resulting in a pixel time of 0.16 seconds. Post-imaging slides were hematoxylin and eosin stained and scanned on a Motic EasyScan (Motic, Xiamen, China) slide scanner.

Pharmacokinetic studies in rat, dog, and monkey (AbbVie, Inc.)

PK studies were conducted in IGS Crl:CD(SD) rats, beagle dogs (Marshall BioResources, North Rose, NY), and cynomolgous monkeys. Rats were administered test item by oral gavage doses of 2 mg/kg in 10% ethanol: 30% PEG-400: 60% Phosal 53 MCT, by weight (PhMCT) at 2 mL/kg, and 10, 30, 100, 200, and 300 mg/kg in 10% ethanol: 30% PEG-400: 60% Phosal 50 PG, by weight (PhPG) at 2 mL/kg. Rats were also administered an IV dose of 1 mg/kg in 10% DMSO: 90% PEG-400 (v/v) (DP400) at 1 mL/kg. Dogs were administered PO and IV doses of 1 mg/kg in DP400 at 0.5 mL/kg and Torpac size 12 oral capsules containing doses of 3, 10, and 30 mg/kg in PhPG at 2 mL/kg. Monkeys were administered PO and IV doses of 1 mg/kg in DP400 at 0.5 mL/kg. Serial blood samples were collected in tubes containing anticoagulant (EDTA or heparin) and centrifuged to collect plasma for LC-MS/MS quantitation. Samples were extracted in acetonitrile and separated on a 50 x 3 mm 2.7 µM Ascentis Express column (Supelco, Bellefonte, PA, USA) and analyzed on a Sciex API5000 (Sciex, Framingham, MA, USA) triple quadrupole mass spectrometer and integrated using Sciex Analyst software. Concentrations for each sample were calculated by least squares linear regression analysis of peak area ratios of spiked plasma standards versus concentration.

Repeat dose exploratory toxicology studies in rat and dog (SRI International)

Toxicology studies in rats were conducted in male and female (n=14, each) Sprague Dawley rats (Charles River Laboratories, Wilmington, MA, USA), 7-9 weeks old, housed 3 per cage and provided Envigo Teklad Certified Global 18% protein rodent diet (2018C) and water ad libitum. Rats were orally administered via gavage 10 mL/kg of BKI-1708 in PhPG vehicle. Dose formulations were prepared up to 1 week prior to use and stored at ambient temperature in the dark. Rats were dosed 30, 75, and 200 mg/kg/day for 14 consecutive days. Animals were checked daily for mortality/morbidity and

for clinical alterations including gross motor and behavioral activity and appearance. Body weights were recorded before start of dosing and twice weekly or daily for animal showing signs weight loss. Animals exhibiting ≥20% weight loss or showing signs of morbidity were euthanized for necropsy. Blood was sampled by retro-orbital sinus or tail vein into K_2EDTA tubes for hematology and clinical chemistry and processed for determination of plasma drug levels via LC-MS/MS using a 50 x 2 mm 5 µM Agilent Polaris 5 C18-A column (Agilent Technologies, Santa Clara, CA, USA) and Waters Xevo TQ-S triple quadrupole mass spectrometer and integrated using MassLynx software. Rats were fasted 12 h prior to necropsy and were anesthetized by isoflurane inhalation and euthanized by exsanguination. Gross examination, organ weights, and histopathologic examination of tissues were evaluated by a board-certified veterinary pathologist.

For toxicology studies in dogs, male and female (n=2, each) Beagle dogs (Marshall Bioresources, North Rose, NY, USA), 7-8 months old, were housed singly in 4 x 6 ft enclosures and provided Envigo Teklad Certified Global 25% protein dog diet (2025C) and water ad libitum. Animals were fasted overnight before dosing and food was returned ~2 h after dosing. Dogs were orally administered gelatin capsules containing 2 mL/kg of BKI-1708 in PhPG vehicle. Dose formulations were prepared and stored at ambient temperature up to 5 days prior to day of use. Dogs were dosed 10, 30, and 50 mg/kg/day for 5 consecutive days. Animals were checked daily for mortality/morbidity and for clinical alterations including gross motor and behavioral activity and appearance. Body weights were recorded before start of dosing and 24 h post final administration. Blood was sampled from cephalic or jugular veins into K_2EDTA tubes for hematology and clinical chemistry and processed for determination of plasma drug levels using a 50 x 2.1 mm 5 µM Kinetex C8 column (Phenomenex, Torrance, CA, USA) and Sciex API4000 (Framingham, MA, USA) triple quadrupole mass spectrometer and integrated using Sciex Analyst software.

Human dosing regimen prediction (based on allometric scaling and BKI-1708 exposure observed with 15 mg/kg QD dosing for 3 days in mice)

Human PK parameters for BKI-1708 were predicted using three methods: allometric scaling of the PK parameter values observed in mouse, rat, dog, and monkey PK studies; in vitro-in vivo extrapolation (IVIVE) from mouse, rat, dog, and non-human primate hepatocyte studies; and IVIVE from mouse, rat, dog, and non-human primate liver microsome studies. Unadjusted allometric scaling and allometric scaling adjusted by maximum lifespan potential, brain weight, or fraction unbound in plasma were used to estimate human clearance. The exponent for simple allometric scaling was 0.71. As a result, by the rule of exponents[102], allometric scaling adjusted by maximum lifespan potential was determined most likely to provide an accurate estimate of human clearance for BKI-1708. With our allometric scaling approach, a plasma clearance of 3.7 L/h and volume of distribution of 104.4 L were estimated for BKI-1708 in humans. For each IVIVE approach, the human hepatic clearance estimates were based on the well-stirred model. With the high degree of BKI-1708 binding in plasma, renal filtration clearance is expected to have a relatively small contribution to total body clearance and it was not included in the total body clearance estimate. The human PK parameters derived from IVIVE from hepatocyte studies and microsome studies are reported in **S8 Table**.

Human PK parameters estimated with allometric scaling and IVIVE approaches were used to predict a daily BKI-1708 dose for treatment of cryptosporidiosis in humans (**S9 Table**). Based on the non-human primate PK study, BKI-1708 was predicted to have an oral bioavailability in humans of 0.8.

BKI-1708 Safety margin estimate

Pharmacokinetic (PK) model development, simulation, and analysis were performed with Phoenix NLME (version 8.3.5, Pharsight, Certara Inc., Princeton, NJ, USA). Compartmental population PK models were fitted to BKI-1708 plasma concentrations observed in BL6 IFN$\gamma^{-/-}$ mice and Sprague Dawley rats that received a single intravenous dose of BKI-

1708 at 3 mg/kg and 1 mg/kg, respectively. A 1-compartment PK structural model with a proportional error model was fitted to the mouse data and the rat data. Both models assumed linear pharmacokinetics. Model selection was based on least squares optimization and visual inspection of residuals for linearity, normality, and homoscedasticity. Mice and rats also received a single oral dose of BKI-1708 at 15 mg/kg and 2 mg/kg, respectively, and PK models were fitted to the plasma concentrations. A 1-compartment model with an additive-multiplicative error model was fitted to the mouse data. A 1-compartment model with a proportional error model was fitted to the rat data. A first-order absorption process was incorporated into each model with extravascular drug administration.

The mouse and rat PK models for oral dosing were used to simulate BKI-1708 plasma concentrations following multiple oral doses. Simulated concentration-time profiles were compared against observed data in mice and rats, and considered to be appropriate when bias was low (i.e., average fold error was between 0.5 and 2) and precision was high (i.e., absolute average fold error was between 0.5 and 2). BKI-1708 exposure (i.e., area under the plasma concentration-time curve (AUC)) was estimated by non-compartmental analysis (NCA) for each simulated BKI-1708 dosing regimen. If models were deemed inappropriate, exposure estimates were determined by NCA using observed BKI-1708 plasma concentrations.

The mouse BKI-1708 dosing regimen at 15 mg/kg QD for 3 days was considered effective as it significantly reduced *Cryptosporidium* oocyst shedding compared to untreated controls. The BKI-1708 exposure was estimated for the efficacious dosing regimen by simulating BKI-1708 concentrations in mice dosed 15 mg/kg QD for 3 days. Safety margins were determined by dividing the average estimated BKI-1708 exposure in each toxicology study (**S10 Table**) by the average estimated BKI-1708 exposure in the mouse efficacy study (15 mg/kg QD for 3 days).

BKI-1708 exposure was estimated in the mouse and 5-day rat toxicology studies by simulating BKI-1708 concentrations for each dosing regimen. Three safety margins were reported for each study: a safety margin considering the full toxicology and efficacy study periods, a margin considering only a 24-hour period following the first dose (AUC_{0-24h}), and a margin considering only a 24-hour period following the last dose (**S11 and S12 Tables**).

In a 5-day toxicology study in dogs and a 14-day study in rats, there was a decrease in the observed BKI-1708 in plasma concentrations following the last doses compared to the first oral doses. As a result of the non-linear pharmacokinetics, BKI-1708 exposure in dogs and the 14-day rat study were not estimated from simulated plasma concentration over time. Instead, NCA was used to estimate the exposure for the first and last dosing days. Both the first and last days included rich sampling of BKI-1708 concentrations in plasma. A linear regression was fit to the exposure during the first and last dosing days and used to interpolate the exposure to BKI-1708 during intermediate dosing days. The exposure to BKI-1708 in each dog/rat in the toxicology studies were estimated as the sum of the exposure estimates for each of the dosing days. As described above for mice and rats, three safety margins were reported (**S13 and S14 Tables**).
