# Supplementary material for: Anti-Cryptosporidium efficacy of BKI-1708, an inhibitor of Cryptosporidium calcium-dependent protein kinase 1
Source: PLoS Negl Trop Dis. 2025 Jul 30;19(7):e0013263. doi: 10.1371/journal.pntd.0013263 (PMC12310023; doi:10.1371/journal.pntd.0013263)
Supplement: S1 Fig — (PDF) [file pntd.0013263.s002.pdf]

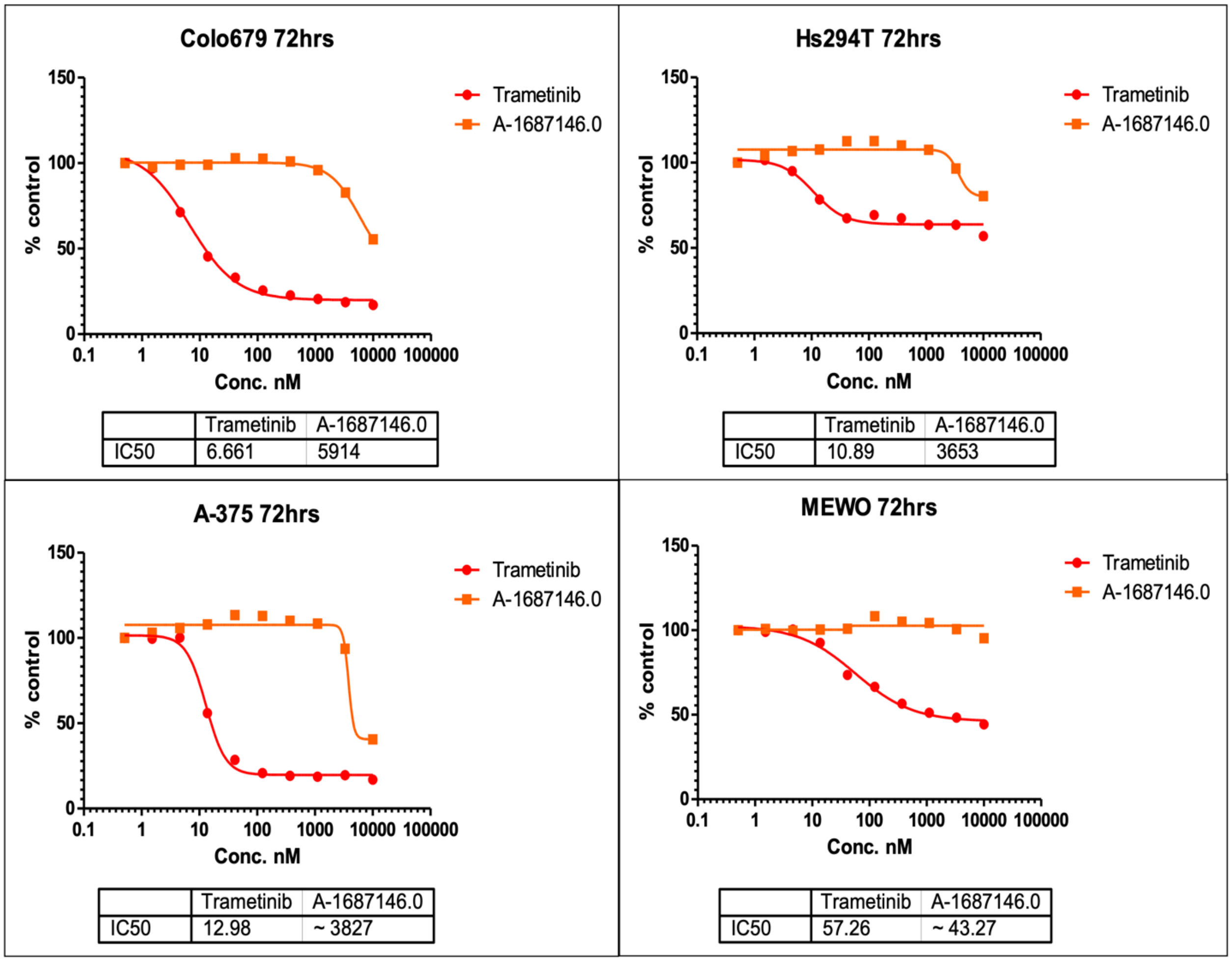

S1 Fig. Cellular proliferation assays to screen for MEK inhibition. BKI-1708 was tested in cellular proliferation assays in 4 carcinoma cell lines (A-375, Hs294T, MEWO, and Colo678) against a clinically approved MEK inhibitor, Trametinib. A-1687146.0 = BKI-1708. The calculated IC_{50} of BKI-1708 in MEWO cells are an artifact of the curve fitting algorithm and does not appear to represent true activity. BKI-1708 showed >95% proliferation relative to untreated controls at all concentrations tested.
