# Supplementary material for: Anti-Cryptosporidium efficacy of BKI-1708, an inhibitor of Cryptosporidium calcium-dependent protein kinase 1
Source: PLoS Negl Trop Dis. 2025 Jul 30;19(7):e0013263. doi: 10.1371/journal.pntd.0013263 (PMC12310023; doi:10.1371/journal.pntd.0013263)
Supplement: S2 Fig — (PDF) [file pntd.0013263.s003.pdf]

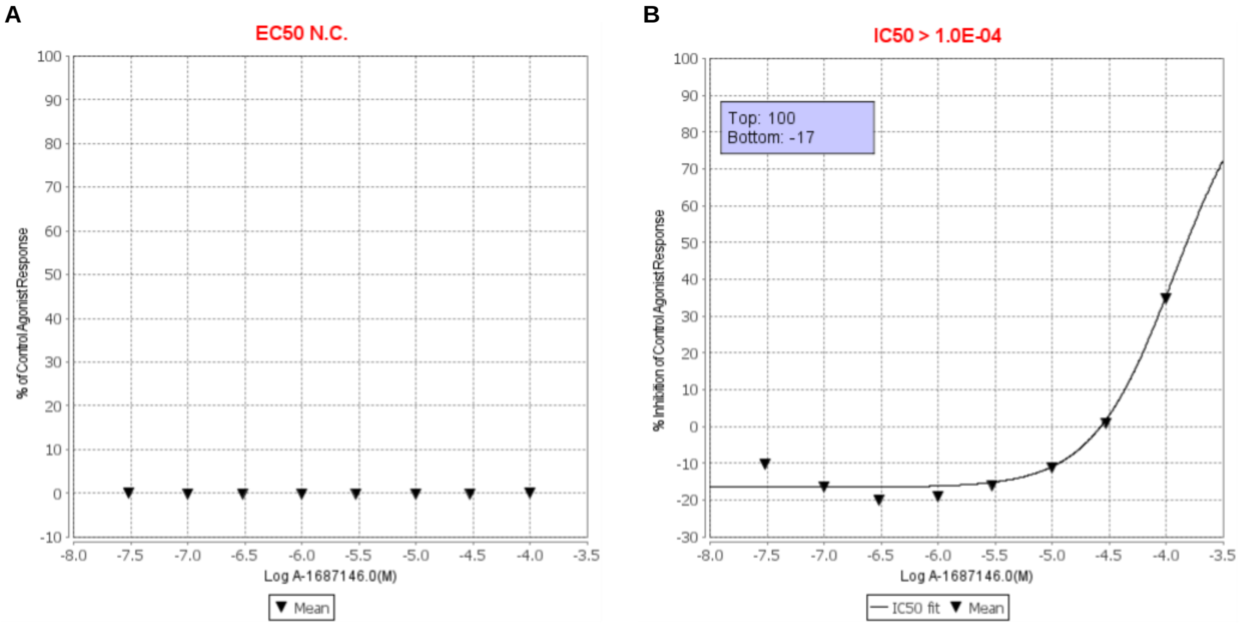

S2 Fig. Cerep cellular and nuclear receptor functional assay: Agonist and antagonist effect of BKI-1708 on PPARУ. To further investigate the potential for PPARУ binding, BKI-1708 was tested at several concentrations for IC_{50} determination in a cellular and nuclear receptor functional assay against agonist and antagonist substrates of PPARУ. BKI-1708 did not show significant activity with concentrations up to 100 µM **(A)** Agonist effect. BKI-1708 exhibited no effect up to 100 µM. Rosiglitazone reference (0.27 µM EC_{50}) **(B)** Antagonist effect. BKI-1708 IC_{50} >100 µM. GW 9662 reference (0.034 µM IC_{50}).
