# Supplementary material for: Anti-Cryptosporidium efficacy of BKI-1708, an inhibitor of Cryptosporidium calcium-dependent protein kinase 1
Source: PLoS Negl Trop Dis. 2025 Jul 30;19(7):e0013263. doi: 10.1371/journal.pntd.0013263 (PMC12310023; doi:10.1371/journal.pntd.0013263)
Supplement: S3 Fig — (PDF) [file pntd.0013263.s004.pdf]

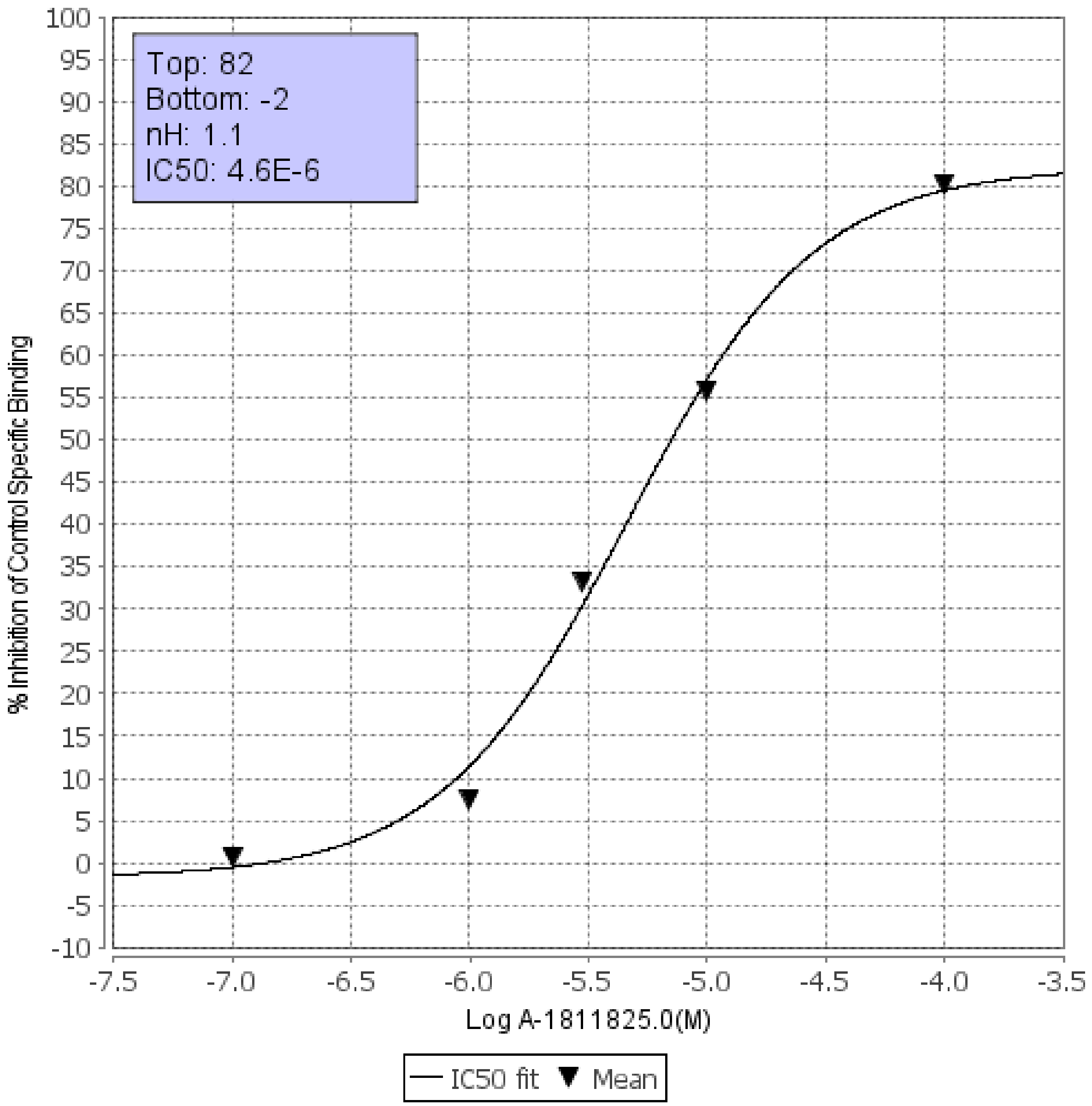

S3 Fig. Cerep binding assay: Agonist effect of BKI-1708 metabolite, M2 on AT_2. Angiotensin-II reference (0.062 nM IC_{50}). A-1811825.0 = M2, IC_{50}: 4.6 μM.
