# Supplementary material for: Anti-Cryptosporidium efficacy of BKI-1708, an inhibitor of Cryptosporidium calcium-dependent protein kinase 1
Source: PLoS Negl Trop Dis. 2025 Jul 30;19(7):e0013263. doi: 10.1371/journal.pntd.0013263 (PMC12310023; doi:10.1371/journal.pntd.0013263)
Supplement: S5 Fig — (PDF) [file pntd.0013263.s006.pdf]

A B

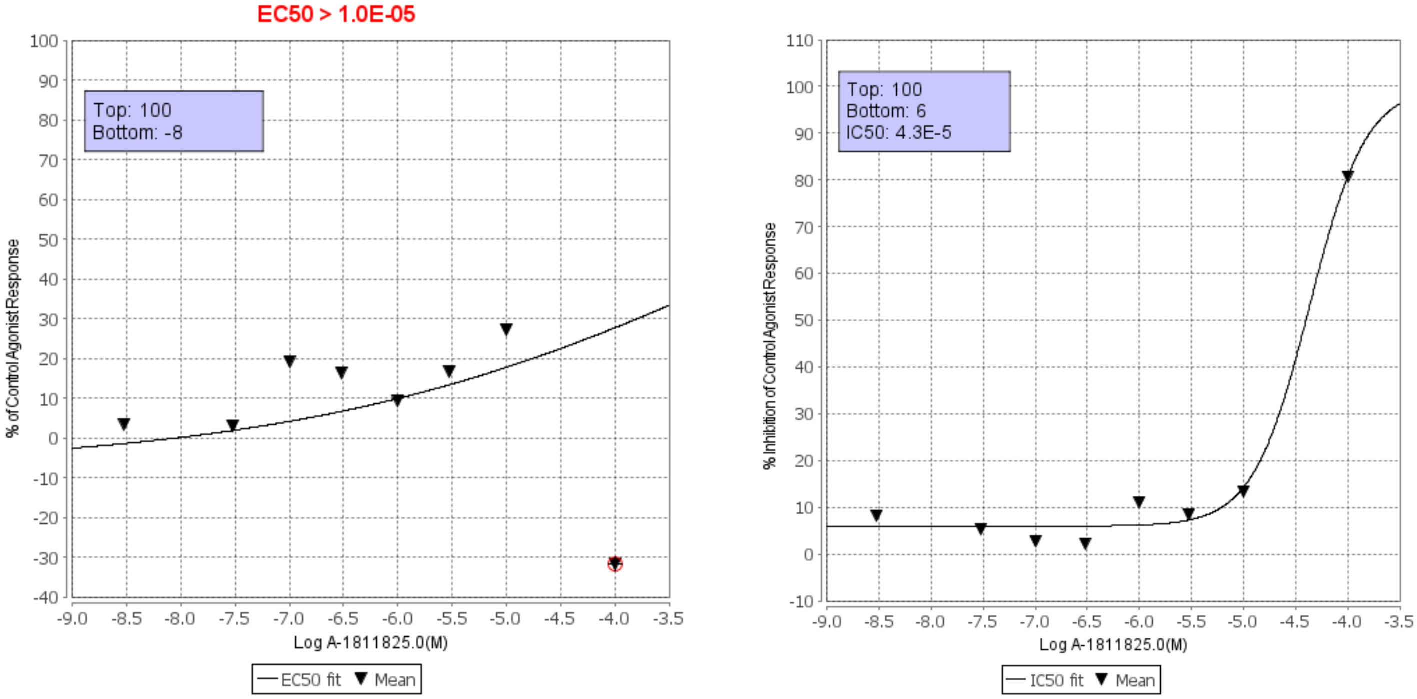

S5 Fig. Cerep cellular and nuclear receptor functional assay: Agonist and antagonist effect of BKI-1708 metabolite, M2 on A_3. (A) Agonist effect. IB-MECA reference (0.75 nM EC_{50}) A-1811825.0 = M2, EC_{50}: >10 μM. **(B)** Antagonist effect. MRS 1220 reference (6.9 nM IC_{50}). M2 IC_{50} 43 μM.
