# Supplementary material for: Anti-Cryptosporidium efficacy of BKI-1708, an inhibitor of Cryptosporidium calcium-dependent protein kinase 1
Source: PLoS Negl Trop Dis. 2025 Jul 30;19(7):e0013263. doi: 10.1371/journal.pntd.0013263 (PMC12310023; doi:10.1371/journal.pntd.0013263)
Supplement: S6 Fig — (PDF) [file pntd.0013263.s007.pdf]

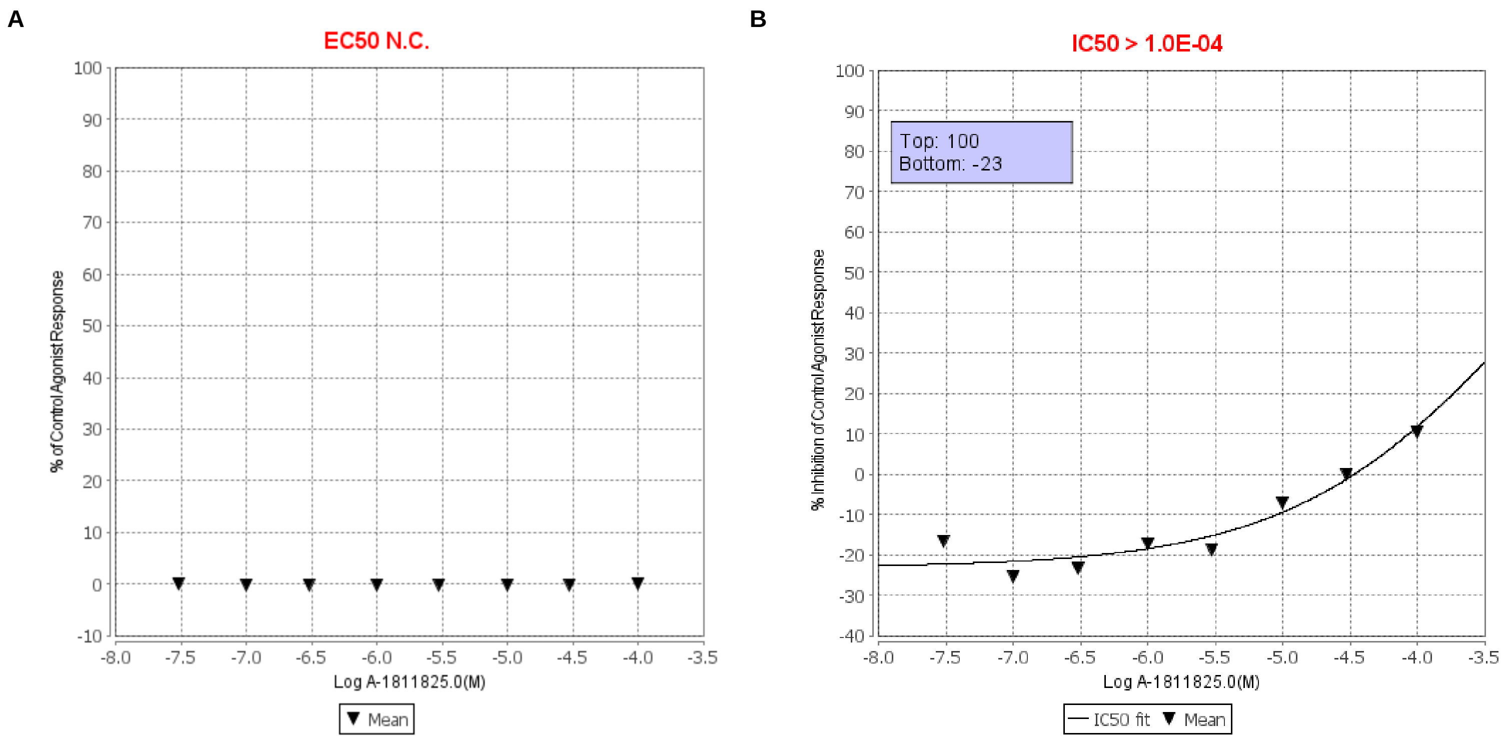

S6 Fig. Cerep cellular and nuclear receptor functional assay: Agonist and antagonist effect of BKI-1708 metabolite, M2 on PPARУ. (A) Agonist effect. Rosiglitazone reference (0.27 μM EC_{50}). A-1811825.0 = M2, exhibited no effect up to 100 μM. **(B)** Antagonist effect. GW 9662 reference (0.034 μM IC_{50}). M2 IC_{50} >100 μM.
