# Supplementary figures and images for: Anti-Cryptosporidium efficacy of BKI-1708, an inhibitor of Cryptosporidium calcium-dependent protein kinase 1
Source: PLoS Negl Trop Dis. 2025 Jul 30;19(7):e0013263. doi: 10.1371/journal.pntd.0013263 (PMC12310023; doi:10.1371/journal.pntd.0013263)

**A**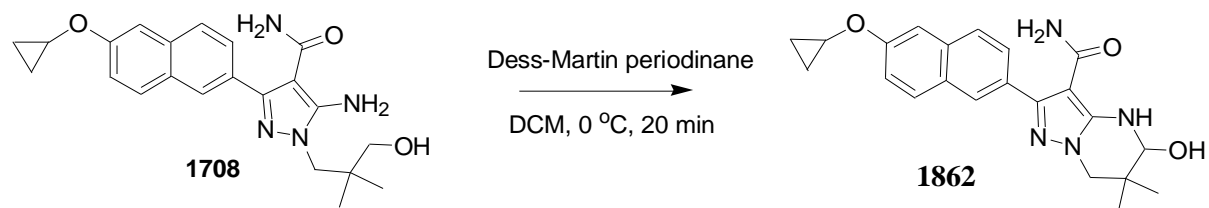**B**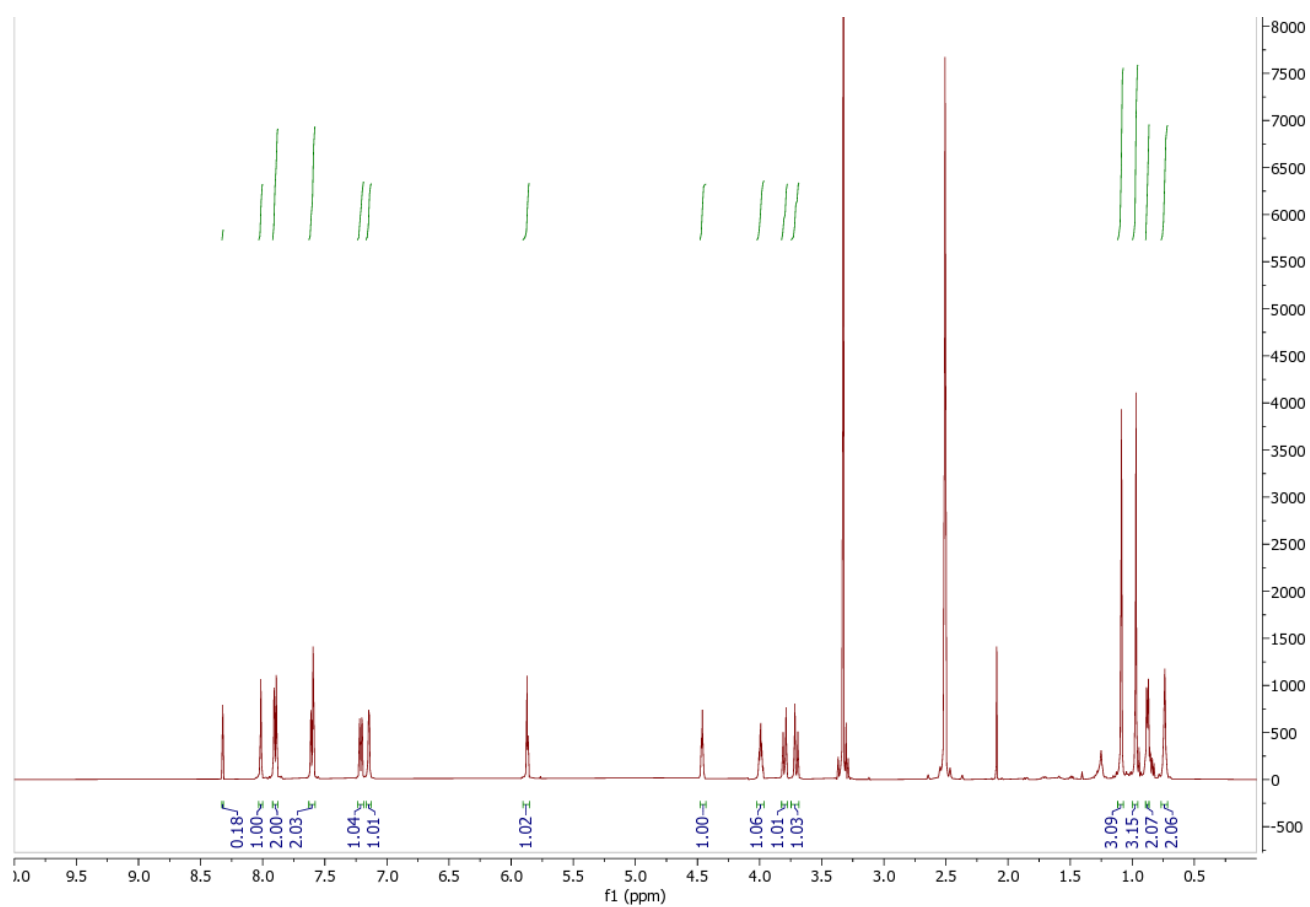

**S8 Fig. Scheme 1. (A)** Synthesis of metabolite M2 from BKI-1708. **(B)**  $^1\text{H}$  NMR (500 MHz,  $\text{DMSO}-d_6$ ).

Supplement: S8 Fig — (PDF) [file pntd.0013263.s009.pdf]
