# Supplementary material for: Anti-Cryptosporidium efficacy of BKI-1708, an inhibitor of Cryptosporidium calcium-dependent protein kinase 1
Source: PLoS Negl Trop Dis. 2025 Jul 30;19(7):e0013263. doi: 10.1371/journal.pntd.0013263 (PMC12310023; doi:10.1371/journal.pntd.0013263)
Supplement: S1 Table — BKI-1708 [M2] plasma exposures before and after final administration. (PDF) [file pntd.0013263.s010.pdf]

S1 Table. Efficacy of BKI-1708 in the IFNγ-KO mouse model of cryptosporidiosis. BKI-1708 [M2] plasma exposures before and after final administration.

Regimen	Mouse	Trough	0.5 h	1 h	2 h
**60 mg/kg QD x 3**	**1**	ND	14.2 [ND]	ND	ND
	**2**	ND	10.4 [ND]	ND	ND
	**3**	ND	14.9 [ND]	ND	ND
**30 mg/kg QD x 3**	**1**	ND	3.7 [7.9]	ND	ND
	**2**	ND	5.4 [14.9]	ND	ND
	**3**	ND	11.6 [14.4]	ND	ND
**15 mg/kg QD x 3**	**1**	ND	3.6 [5.2]	ND	ND
	**2**	ND	7.0 [9.9]	ND	ND
	**3**	ND	5.3 [12.0]	ND	ND
**8 mg/kg QD x 3**	**1**	ND	4.6 [2.4]	ND	ND
	**2**	ND	3.8 [3.2]	ND	ND
	**3**	ND	1.7 [2.1]	ND	ND
**15 mg/kg QD x 3**	**1**	0.0 [0.1]	1.5 [0.9]	1.9 [3.0]	2.0 [5.8]
	**2**	0.0 [0.2]	6.9 [1.1]	6.6 [4.7]	4.9 [10.5]
	**3**	0.0 [0.1]	2.8 [3.5]	2.4 [7.6]	1.1 [8.9]
**15 mg/kg QD x 2**	**1**	0.0 [0.0]	ND	2.6 [7.8]	1.6 [10.1]
	**2**	0.0 [0.0]	2.7 [3.4]	2.4 [6.1]	2.0 [11.8]
	**3**	0.0 [0.0]	1.7 [2.5]	2.5 [7.9]	1.5 [11.4]
**15 mg/kg QD x 1**	**1**	ND	3.5 [4.3]	2.8 [7.8]	1.4 [8.8]
	**2**	ND	2.5 [3.8]	2.4 [6.2]	1.8 [10.4]
	**3**	ND	1.6 [2.8]	2.2 [4.2]	2.5 [11.2]
**60 mg/kg QD x 1**	**1**	ND	1.1 [0.9]	0.6 [1.4]	1.3 [4.3]
	**2**	ND	5.0 [8.0]	4.4 [13.1]	3.3 [18.7]
	**3**	ND	7.2 [3.6]	7.8 [8.2]	4.3 [12.3]
**30 mg/kg QD x 1**	**1**	ND	4.9 [3.3]	4.3 [8.5]	2.3 [12.3]
	**2**	ND	5.0 [6.3]	3.1 [9.0]	1.7 [10.9]
	**3**	ND	2.2 [2.9]	3.1 [6.9]	3.9 [13.5]

BKI-1708 [M2] values are reported in μM concentrations.
