# Supplementary material for: Anti-Cryptosporidium efficacy of BKI-1708, an inhibitor of Cryptosporidium calcium-dependent protein kinase 1
Source: PLoS Negl Trop Dis. 2025 Jul 30;19(7):e0013263. doi: 10.1371/journal.pntd.0013263 (PMC12310023; doi:10.1371/journal.pntd.0013263)
Supplement: S2 Table — (PDF) [file pntd.0013263.s011.pdf]

S2 Table. BKI-1708 and M2 metabolite activity against the bioprofiling panel of 20 common liability targets (37 functional assays).

Assay	Readout	Value	BKI-1708	M2
**5-HT1A**	**agonist**	**EC_{50}**	> 10	> 10
**5-HT1A**	**antagonist**	**IC_{50}**	> 10	> 10
**5-HT2A**	**agonist**	**EC_{50}**	> 10	> 10
**5-HT2A**	**antagonist**	**IC_{50}**	> 10	> 10
**5-HT2B**	**agonist**	**EC_{50}**	> 10	> 10
**5-HT2B**	**antagonist**	**IC_{50}**	> 10	> 10
**A1**	**agonist**	**EC_{50}**	> 10	> 10
**A1**	**antagonist**	**IC_{50}**	> 10	> 10
**Alpha1A**	**agonist**	**EC_{50}**	> 10	> 10
**Alpha1A**	**antagonist**	**IC_{50}**	> 10	> 10
**Alpha2A**	**agonist**	**EC_{50}**	> 10	> 10
**Alpha2A**	**antagonist**	**IC_{50}**	> 10	> 10
**B2**	**agonist**	**EC_{50}**	> 10	> 10
**B2**	**antagonist**	**IC_{50}**	> 10	> 10
**Beta1**	**agonist**	**EC_{50}**	> 10	> 10
**Beta1**	**antagonist**	**IC_{50}**	> 10	> 10
**Cav1.2 (L-type)**	**agonist**	**EC_{50}**	> 10	> 10
**Cav1.2 (L-type)**	**antagonist**	**IC_{50}**	> 10	8.1
**CB1**	**agonist**	**EC_{50}**	> 10	> 10
**CB1**	**antagonist**	**IC_{50}**	> 10	> 10
**CHO**	**agonist**	**EC_{50}**	> 10	> 10
**D2L**	**agonist**	**EC_{50}**	> 10	> 10
**D2L**	**antagonist**	**IC_{50}**	> 10	> 10
**ETA**	**agonist**	**EC_{50}**	> 10	> 10
**ETA**	**antagonist**	**IC_{50}**	> 10	> 10
**ETB**	**agonist**	**EC_{50}**	> 10	> 10
**ETB**	**antagonist**	**IC_{50}**	> 10	> 10
**H1**	**agonist**	**EC_{50}**	> 10	> 10
**H1**	**antagonist**	**IC_{50}**	> 10	> 10
**M2**	**agonist**	**EC_{50}**	> 10	> 10
**M2**	**antagonist**	**IC_{50}**	> 10	> 10
**Opioid mu**	**agonist**	**EC_{50}**	> 10	> 10
**Opioid mu**	**antagonist**	**IC_{50}**	> 10	> 10
**P2Y**	**antagonist**	**IC_{50}**	> 10	> 10
**PDE3A**	**antagonist**	**IC_{50}**	> 10	> 10
**PDE4B2**	**antagonist**	**IC_{50}**	> 10	> 10
**PPARgamma**	**agonist**	**EC_{50}**	> 10	> 10

Compounds were tested using 6 pt dilutions starting at 10 μM to determine IC_{50}/EC_{50} values. Values are reported in μM concentrations.
