# Supplementary material for: Anti-Cryptosporidium efficacy of BKI-1708, an inhibitor of Cryptosporidium calcium-dependent protein kinase 1
Source: PLoS Negl Trop Dis. 2025 Jul 30;19(7):e0013263. doi: 10.1371/journal.pntd.0013263 (PMC12310023; doi:10.1371/journal.pntd.0013263)
Supplement: S3 Table — (PDF) [file pntd.0013263.s012.pdf]

S3 Table. BKI-1708 activity against the Cerep panel of 71 common liability targets: Binding assays. BKI-1708 was tested at a concentration of 10 µM for its potential to interfere with radioactively labeled native ligands, and compound binding was defined as percent inhibition of control specific binding with a response of ≥50% considered a significant effect. BKI-1708 exhibited significant inhibition (52.8%) of the agonist radioligand for the nuclear receptor protein, peroxisome proliferator-activated receptor gamma (PPARУ), which regulates lipid homeostasis.

		% inhibition of control specific binding		
**Assay**	**Radioligand**	**1st replicate**	**2nd replicate**	**Mean**
**A1**	**antagonist**	9.2	15.4	12.3
**A2A**	**agonist**	0.3	1.1	0.7
**A2B**	**antagonist**	6.4	10.7	8.6
**A3**	**agonist**	36.4	37.7	37.1
**α1A**	**antagonist**	7	9	8.0
**α1B**	**antagonist**	-6.5	0.9	-2.8
**α2A**	**antagonist**	7.8	2.4	5.1
**α2B**	**antagonist**	-14.4	20.3	3.0
**β1**	**agonist**	3.6	5.1	4.4
**β2**	**antagonist**	-5.6	0.4	-2.6
**AT1**	**antagonist**	16.6	12.2	14.4
**AT2**	**agonist**	-2.6	-6.4	-4.5
**BZD**	**agonist**	44.8	45.2	45.0
**B2**	**agonist**	-12.8	-17.9	-15.4
**CGRP**	**agonist**	-10.9	-12.8	-11.9
**CB1**	**agonist**	-9.9	-0.2	-5.1
**CB2**	**agonist**	17.7	17.9	17.8
**CCK1 (CCKA)**	**agonist**	-0.2	3.8	1.8
**D1**	**antagonist**	-0.4	1.5	0.6
**D2S**	**antagonist**	15.3	2.6	9.0
**ETA**	**agonist**	-1	4.7	1.9
**ETB**	**agonist**	-0.9	0.9	0.0
**GABAA1**	**agonist**	5.6	7.5	6.6
**NMDA**	**antagonist**	0.8	-2.1	-0.7
**TNF-α**	**agonist**	3.8	-4.1	-0.2
**H1**	**antagonist**	7.4	3.2	5.3
**H2**	**antagonist**	-9	-22.8	-15.9
**MT2 (ML1B)**	**agonist**	-6.5	-3.9	-5.2
**MAO-A**	**antagonist**	14.6	11.3	13.0
**motilin**	**agonist**	-15.5	-13.2	-14.4
**M1**	**antagonist**	-6.2	12.9	3.4
**M2**	**antagonist**	-2.2	8.3	3.1
**M3**	**antagonist**	-10.1	-7.6	-8.9
**M4**	**antagonist**	-35.8	-37.2	-36.5

**M5**	**antagonist**	-10.2	-2.8	-6.5
**NK1**	**agonist**	15.9	-1.8	7.1
**NK2**	**agonist**	10.5	18.9	14.7
**Y1**	**agonist**	-10.7	-3.9	-7.3
**δ (DOP)**	**agonist**	14.1	3.9	9.0
**kappa**	**agonist**	24.5	14.8	19.7
**μ (MOP)**	**agonist**	9.7	16.2	13.0
**PPARγ**	**agonist**	54.3	51.4	**52.9**
**PCP**	**antagonist**	1.6	-10.6	-4.5
**EP2**	**agonist**	44	47.1	45.6
**P2X**	**agonist**	-2.8	-10.4	-6.6
**5-HT1A**	**agonist**	1.1	15.8	8.5
**5-HT1B**	**antagonist**	-2	-11.3	-6.7
**5-HT2A**	**antagonist**	7.7	1.3	4.5
**5-HT2B**	**agonist**	20.6	28	24.3
**5-HT2C**	**antagonist**	13.9	24.4	19.2
**5-HT3**	**antagonist**	6.7	1.8	4.3
**5-HT4e**	**antagonist**	0.3	9	4.7
**5-HT7**	**agonist**	-15.2	-25.7	-20.5
**sigma (non-selective)**	**agonist**	7.1	22.9	15.0
**GR**	**agonist**	14	8.2	11.1
**Estrogen ER alpha**	**agonist**	8.8	1.1	5.0
**AR**	**agonist**	-2.4	-14.7	-8.6
**BZDp (TSPO)**	**antagonist**	4.3	13.4	8.9
**UT**	**agonist**	-10	-1.5	-5.8
**VPAC1 (VIP1)**	**agonist**	-17.4	-12	-14.7
**V1a**	**agonist**	14.8	6.1	10.5
**Ca2+ channel (L, dihydropyridine site)**	**antagonist**	-0.6	-8.5	-4.6
**Ca2+ channel (L, diltiazem site) (benzothiazepines)**	**antagonist**	11.3	12.9	12.1
**KATP channel**	**antagonist**	2.3	-3.3	-0.5
**SKCa channel**	**antagonist**	2.5	-8.7	-3.1
**Na+ channel (site 2)**	**antagonist**	13	24.8	18.9
**Cl- channel (GABA-gated)**	**antagonist**	14.6	12.1	13.4
**norepinephrine transporter**	**antagonist**	8.7	4.2	6.5
**dopamine transporter**	**antagonist**	12.3	4	8.2
**GABA transporter**	**antagonist**	-2.8	-10.8	-6.8
**5-HT transporter**	**antagonist**	-4.7	1.4	-1.7

BKI-1708 was screened at a concentration of 10 μM. Results showing >50% inhibition are considered to represent significant effects.
