# Supplementary material for: Anti-Cryptosporidium efficacy of BKI-1708, an inhibitor of Cryptosporidium calcium-dependent protein kinase 1
Source: PLoS Negl Trop Dis. 2025 Jul 30;19(7):e0013263. doi: 10.1371/journal.pntd.0013263 (PMC12310023; doi:10.1371/journal.pntd.0013263)
Supplement: S4 Table — (PDF) [file pntd.0013263.s013.pdf]

**S4 Table. BKI-1708 activity against the Cerep panel of 71 common liability targets: Enzyme and Uptake assays.**

| Enzyme/Uptake Assay | 1st replicate | 2nd replicate | Mean |
|---|---|---|---|
| **COX1** | 27.4 | 18.4 | 22.9 |
| **COX2** | -0.5 | -2.6 | -1.6 |
| **PDE3A** | -6.1 | 9.3 | 1.6 |
| **ACE** | -7.5 | -9.9 | -8.7 |
| **acetylcholinesterase** | 5.6 | -0.5 | 2.6 |
| **MAO-B** | 2.3 | 0.8 | 1.6 |
| **ATPase (Na+/K+)** | -4.7 | -6.2 | -5.5 |

*BKI-1708 was screened at a concentration of 10 µM. Results showing >50% inhibition are considered to represent significant effects.*
