# Supplementary material for: Anti-Cryptosporidium efficacy of BKI-1708, an inhibitor of Cryptosporidium calcium-dependent protein kinase 1
Source: PLoS Negl Trop Dis. 2025 Jul 30;19(7):e0013263. doi: 10.1371/journal.pntd.0013263 (PMC12310023; doi:10.1371/journal.pntd.0013263)
Supplement: S5 Table — (PDF) [file pntd.0013263.s014.pdf]

S5 Table. BKI-1708 metabolite, M2 activity against the Cerep panel of 71 common liability targets: Binding assays. The Cerep radioligand binding assay was conducted at a 10 μM concentration and identified agonist radioligand displacement for the following targets: A3 adenosine receptor (72.6% inhibition), angiotensin type II (AT2) receptor (50.7%), PPARУ (70%), and 5-hydroxytryptamine 2B (5-HT2B) receptor (57.1%). Displacement of the antagonist radioligand for the GABA-gated Cl- channel (GABAA) was also observed (50.5%).

		**% inhibition of control specific binding**		
**Assay**	**Radioligand**	**1st replicate**	**2nd replicate**	**Mean**
**A1**	**antagonist**	13	8.2	10.6
**A2A**	**agonist**	1.8	4.1	3.0
**A2B**	**antagonist**	12.7	1.8	7.3
**A3**	**agonist**	69.6	75.6	**72.6**
**α1A**	**antagonist**	-3.7	-0.5	-2.1
**α1B**	**antagonist**	12.6	21.1	16.9
**α2A**	**antagonist**	6.6	7.6	7.1
**α2B**	**antagonist**	-16.2	-9.9	-13.1
**β1**	**agonist**	-10.9	-2.2	-6.6
**β2**	**antagonist**	4.2	5.3	4.8
**AT1**	**antagonist**	15	24.7	19.9
**AT2**	**agonist**	49.1	52.3	**50.7**
**BZD**	**agonist**	18.7	36.2	27.5
**B2**	**agonist**	-12.4	-16.9	-14.7
**CGRP**	**agonist**	-15.6	-14	-14.8
**CB1**	**agonist**	-2.2	0.4	-0.9
**CB2**	**agonist**	48.3	40.9	44.6
**CCK1 (CCKA)**	**agonist**	6.1	8.6	7.4
**D1**	**antagonist**	6.7	34	20.4
**D2S**	**antagonist**	-2.6	5.9	1.7
**ETA**	**agonist**	6.3	4.9	5.6
**ETB**	**agonist**	-6.6	6.1	-0.3
**GABAA1**	**agonist**	0.9	2.3	1.6
**NMDA**	**antagonist**	7.6	6.2	6.9
**TNF-α**	**agonist**	7.1	-2.2	2.5
**H1**	**antagonist**	17	4.5	10.8
**H2**	**antagonist**	-2.5	-3.9	-3.2
**MT2 (ML1B)**	**agonist**	11.4	7.4	9.4
**MAO-A**	**antagonist**	15.4	8.9	12.2
**motilin**	**agonist**	-2.2	-17.5	-9.9
**M1**	**antagonist**	-7	3.1	-2.0
**M2**	**antagonist**	-0.2	-0.4	-0.3
**M3**	**antagonist**	-9.9	-11.2	-10.6
**M4**	**antagonist**	-31.3	-42.6	-37.0

**M5**	**antagonist**	-4.7	-0.2	-2.5
**NK1**	**agonist**	6.4	-5.6	0.4
**NK2**	**agonist**	42.3	36.2	39.3
**Y1**	**agonist**	-9.3	6.7	-1.3
**δ (DOP)**	**agonist**	13.1	7	10.1
**kappa**	**agonist**	45.8	34.3	40.1
**μ (MOP)**	**agonist**	19.7	17.7	18.7
**PPARγ**	**agonist**	73	67.1	**70.1**
**PCP**	**antagonist**	-3.4	-7.2	-5.3
**EP2**	**agonist**	25.1	22.9	24.0
**P2X**	**agonist**	-2.2	-15.4	-8.8
**5-HT1A**	**agonist**	-13.5	-9.9	-11.7
**5-HT1B**	**antagonist**	-6.1	-8	-7.1
**5-HT2A**	**antagonist**	11.9	9.5	10.7
**5-HT2B**	**agonist**	55.9	58.3	**57.1**
**5-HT2C**	**antagonist**	11.3	8.9	10.1
**5-HT3**	**antagonist**	5	8.9	7.0
**5-HT4e**	**antagonist**	0	-0.4	-0.2
**5-HT7**	**agonist**	-16	21	2.5
**sigma (non-selective)**	**agonist**	16.4	26.3	21.4
**GR**	**agonist**	-4.1	3.7	-0.2
**Estrogen ER alpha**	**agonist**	-1.6	-2.1	-1.9
**AR**	**agonist**	-17.5	-11.1	-14.3
**BZDp (TSPO)**	**antagonist**	33	29.3	31.2
**UT**	**agonist**	22.2	15.6	18.9
**VPAC1 (VIP1)**	**agonist**	-22	-20.5	-21.3
**V1a**	**agonist**	8.2	2.6	5.4
**Ca2+ channel (L, dihydropyridine site)**	**antagonist**	15.9	8.9	12.4
**Ca2+ channel (L, diltiazem site) (benzothiazepines)**	**antagonist**	7.6	5.4	6.5
**KATP channel**	**antagonist**	2	3.3	2.7
**SKCa channel**	**antagonist**	-9.7	-12.2	-11.0
**Na+ channel (site 2)**	**antagonist**	37.4	33	35.2
**Cl- channel (GABA-gated)**	**antagonist**	47.7	53.4	**50.6**
**norepinephrine transporter**	**antagonist**	12.8	6	9.4
**dopamine transporter**	**antagonist**	6.3	11.5	8.9
**GABA transporter**	**antagonist**	-12.1	-12.9	-12.5
**5-HT transporter**	**antagonist**	-1.9	0.8	-0.6

BKI-1708 was screened at a concentration of 10 μM. Results showing >50% inhibition are considered to represent significant effects.
