# Supplementary material for: Anti-Cryptosporidium efficacy of BKI-1708, an inhibitor of Cryptosporidium calcium-dependent protein kinase 1
Source: PLoS Negl Trop Dis. 2025 Jul 30;19(7):e0013263. doi: 10.1371/journal.pntd.0013263 (PMC12310023; doi:10.1371/journal.pntd.0013263)
Supplement: S6 Table — (PDF) [file pntd.0013263.s015.pdf]

S6 Table. BKI-1708 metabolite, M2 activity against the Cerep panel of 71 common liability targets: Enzyme and Uptake assays.

Enzyme/Uptake Assay	1st replicate	2nd replicate	Mean
**COX1**	26.2	20.5	23.4
**COX2**	-1.7	1.4	-0.2
**PDE3A**	5.9	16.6	11.3
**ACE**	-5.8	-4.3	-5.1
**acetylcholinesterase**	-16.5	-15.5	-16.0
**MAO-B**	4.1	6.9	5.5
**ATPase (Na+/K+)**	-9.2	16.5	3.7

BKI-1708 was screened at a concentration of 10 µM. Results showing >50% inhibition are considered to represent significant effects.
