# Supplementary material for: Anti-Cryptosporidium efficacy of BKI-1708, an inhibitor of Cryptosporidium calcium-dependent protein kinase 1
Source: PLoS Negl Trop Dis. 2025 Jul 30;19(7):e0013263. doi: 10.1371/journal.pntd.0013263 (PMC12310023; doi:10.1371/journal.pntd.0013263)
Supplement: S7 Table — M2 plasma exposures before and after final administration. (PDF) [file pntd.0013263.s016.pdf]

S7 Table. Efficacy of M2 in the IFNγ-KO mouse model of cryptosporidiosis. M2 plasma exposures before and after final administration.

Regimen	Mouse	Trough	0.5 h	1 h	2 h
**30 mg/kg QD x 3**	**1**	0.5	6.7	5.5	7.9
	**2**	0.3	4.6	3.7	7.0
	**3**	0.2	11.6	13.1	6.4
**15 mg/kg QD x 3**	**1**	0.2	6.2	6.9	4.3
	**2**	0.2	7.8	5.5	4.6
	**3**	0.2	5.8	7.9	6.3
**8 mg/kg QD x 3**	**1**	0.1	2.1	2.3	3.2
	**2**	0.1	3.0	2.6	2.7
	**3**	0.1	4.0	4.0	2.2

M2 values are reported in µM concentrations.
