# Supplementary material for: Anti-Cryptosporidium efficacy of BKI-1708, an inhibitor of Cryptosporidium calcium-dependent protein kinase 1
Source: PLoS Negl Trop Dis. 2025 Jul 30;19(7):e0013263. doi: 10.1371/journal.pntd.0013263 (PMC12310023; doi:10.1371/journal.pntd.0013263)
Supplement: S8 Table — (PDF) [file pntd.0013263.s017.pdf]

S8 Table. Predicted human BKI-1708 PK parameters and half-life by allometric scaling or in vitro to in vivo extrapolation.

	Plasma clearance (L/h)	Volume of distribution (L)	Half-life (h)
**Allometric scaling**	3.7	104.4	19.6
**Hepatocyte IVIVE**	15.3	(-)	4.7*
**Microsomal IVIVE**	8.5	(-)	8.5*

**Assumed volume of distribution = 104.4 L based on allometric scaling results. IVIVE: in vitro to in vivo extrapolation.*
