# Supplementary material for: Anti-Cryptosporidium efficacy of BKI-1708, an inhibitor of Cryptosporidium calcium-dependent protein kinase 1
Source: PLoS Negl Trop Dis. 2025 Jul 30;19(7):e0013263. doi: 10.1371/journal.pntd.0013263 (PMC12310023; doi:10.1371/journal.pntd.0013263)
Supplement: S9 Table — (PDF) [file pntd.0013263.s018.pdf]

S9 Table. Predicted human BKI-1708 dosing in 70 kg human based on effective mouse BKI-1708 exposure.

	Daily dose (mg)
**Allometric scaling**	18.0
**Hepatocyte IVIVE**	75.0
**Microsomal IVIVE**	45.0

IVIVE: in vitro to in vivo extrapolation.
