# Supplementary material for: Anti-Cryptosporidium efficacy of BKI-1708, an inhibitor of Cryptosporidium calcium-dependent protein kinase 1
Source: PLoS Negl Trop Dis. 2025 Jul 30;19(7):e0013263. doi: 10.1371/journal.pntd.0013263 (PMC12310023; doi:10.1371/journal.pntd.0013263)
Supplement: S10 Table — (PDF) [file pntd.0013263.s019.pdf]

S10 Table. List of toxicology studies in mice, rats, and dogs.

Animal	Low dose	Middle dose	High dose
**Mouse**	100 mg/kg QD for 7 days	200 mg/kg QD for 7 days	200 mg/kg BID for 5 days
**Rat**	30 mg/kg QD for 5 days and 14 days	75 mg/kg QD for 5 days and 14 days	200 mg/kg QD for 5 days and 14 days
**Dog**	10 mg/kg QD for 5 days	30 mg/kg QD for 5 days	50 mg/kg QD for 5 days

QD: once daily; BID: twice daily.
