# Supplementary material for: Anti-Cryptosporidium efficacy of BKI-1708, an inhibitor of Cryptosporidium calcium-dependent protein kinase 1
Source: PLoS Negl Trop Dis. 2025 Jul 30;19(7):e0013263. doi: 10.1371/journal.pntd.0013263 (PMC12310023; doi:10.1371/journal.pntd.0013263)
Supplement: S11 Table — (PDF) [file pntd.0013263.s020.pdf]

S11 Table. Safety margin for BKI-1708 with 5-7 day mouse study.

Dose regimen	AUC over study period	AUC 0-24h	AUC on last day
**100 mg/kg QD for 7 days**	54.8	23.5	23.5
**200 mg/kg QD for 7 days**	109.5	46.9	46.9
**200 mg/kg BID for 5 days**	156.3	93.9	93.9

QD: once daily; BID: twice daily; AUC: area-under-curve
