# Supplementary material for: Anti-Cryptosporidium efficacy of BKI-1708, an inhibitor of Cryptosporidium calcium-dependent protein kinase 1
Source: PLoS Negl Trop Dis. 2025 Jul 30;19(7):e0013263. doi: 10.1371/journal.pntd.0013263 (PMC12310023; doi:10.1371/journal.pntd.0013263)
Supplement: S13 Table — (PDF) [file pntd.0013263.s022.pdf]

S13 Table. Safety margin for BKI-1708 with 14-day rat study.

Dose regimen	AUC over study period	AUC 0-24h	AUC on last day
**30 mg/kg QD**	36.1	7.7	8.9
**75 mg/kg QD**	89.9	21.9	16.6
**200 mg/kg QD**	107.0	26.8	18.9

QD: once daily; AUC: area-under-curve
