# Supplementary material for: Anti-Cryptosporidium efficacy of BKI-1708, an inhibitor of Cryptosporidium calcium-dependent protein kinase 1
Source: PLoS Negl Trop Dis. 2025 Jul 30;19(7):e0013263. doi: 10.1371/journal.pntd.0013263 (PMC12310023; doi:10.1371/journal.pntd.0013263)
Supplement: S14 Table — (PDF) [file pntd.0013263.s023.pdf]

**S14 Table. Safety margin for BKI-1708 with 5-day dog study.**

| Dose regimen | AUC over study period | AUC 0-24h | AUC on last day |
|---|---|---|---|
| **10 mg/kg QD** | 40.8 | 32.4 | 16.6 |
| **30 mg/kg QD** | 90.8 | 76.3 | 32.6 |
| **50 mg/kg QD** | 90.2 | 80.1 | 28.1 |

*QD: once daily; AUC: area-under-curve*
